# Admixture mapping reveals the association between Native American ancestry at 3q13.11 and reduced risk of Alzheimer’s disease in Caribbean Hispanics

**DOI:** 10.1186/s13195-021-00866-9

**Published:** 2021-07-03

**Authors:** Andréa R. V. R. Horimoto, Diane Xue, Timothy A. Thornton, Elizabeth E. Blue

**Affiliations:** 1 Department of Biostatistics, University of Washington, Seattle, WA USA; 2 Institute for Public Health Genetics, University of Washington, Seattle, WA USA; 3 Division of Medical Genetics, University of Washington, BOX 357720, Seattle, WA 98195-7720 USA

**Keywords:** Admixture mapping, Alzheimer’s disease, Local ancestry, Genetic architecture, Genome scan, Multi-omics

## Abstract

**Background:**

Genetic studies have primarily been conducted in European ancestry populations, identifying dozens of loci associated with late-onset Alzheimer’s disease (AD). However, much of AD’s heritability remains unexplained; as the prevalence of AD varies across populations, the genetic architecture of the disease may also vary by population with the presence of novel variants or loci.

**Methods:**

We conducted genome-wide analyses of AD in a sample of 2565 Caribbean Hispanics to better understand the genetic contribution to AD in this population. Statistical analysis included both admixture mapping and association testing. Evidence for differential gene expression within regions of interest was collected from independent transcriptomic studies comparing AD cases and controls in samples with primarily European ancestry.

**Results:**

Our genome-wide association study of AD identified no loci reaching genome-wide significance. However, a genome-wide admixture mapping analysis that tests for association between a haplotype’s ancestral origin and AD status detected a genome-wide significant association with chromosome 3q13.11 (103.7–107.7Mb, *P* = 8.76E−07), driven by a protective effect conferred by the Native American ancestry (OR = 0.58, 95%CI = 0.47−0.73). Within this region, two variants were significantly associated with AD after accounting for the number of independent tests (rs12494162, *P* = 2.33E−06; rs1731642, *P* = 6.36E−05). The significant admixture mapping signal is composed of 15 haplotype blocks spanning 5 protein-coding genes (*ALCAM*, *BBX*, *CBLB*, *CCDC54*, *CD47*) and four brain-derived topologically associated domains, and includes markers significantly associated with the expression of *ALCAM*, *BBX*, *CBLB*, and *CD47* in the brain. *ALCAM* and *BBX* were also significantly differentially expressed in the brain between AD cases and controls with European ancestry.

**Conclusion:**

These results provide multiethnic evidence for a relationship between AD and multiple genes at 3q13.11 and illustrate the utility of leveraging genetic ancestry diversity via admixture mapping for new insights into AD.

**Supplementary Information:**

The online version contains supplementary material available at 10.1186/s13195-021-00866-9.

## Background

Late-onset Alzheimer’s disease (AD) is a leading cause of death in the USA, affecting approximately 1 in 10 Americans over the age of 65 years, with prevalence expected to double by 2050 [1]. Heritability estimates for AD range from 58 to 79% [2]; despite this strong genetic component, much of the underlying genetic variance remains to be explained [3]. Although the *APOE* ε4 allele is the strongest common genetic risk factor for AD [4, 5], dozens of loci have been associated with AD via genome-wide association studies (GWAS) [6, 7]. However, similar to other complex diseases, the vast majority of genetic discoveries for AD have been from GWAS performed in samples with predominantly European ancestry [8]. Much less is known about AD genetics across diverse populations, and in particular African Americans and Hispanic/Latino Americans who have increased risk of AD compared to European Americans [9–11].

Admixture mapping is a powerful alternative approach to GWAS for gene-mapping in recently admixed populations. Unlike widely used GWAS approaches that treat genetic ancestry differences as potential confounders in the analysis, admixture mapping leverages genetic ancestry differences [12–15]. With admixture mapping, regions of the genome with unusual local ancestry patterns relative to genome-wide averages are tested for association with a phenotype [16]. Admixture mapping is most powerful when both disease risk and trait-locus allele frequencies differ across groups, and it can be viewed as a complement to GWAS [17, 18].

Here, we performed genome scans of AD using both GWAS and admixture mapping approaches to identify regions associated with AD in Caribbean Hispanics, an admixed population with European, Native American, and African ancestry [19, 20]. Admixture mapping identified a genome-wide significant association between AD and Native American ancestry on 3q13.11, while GWAS identified no loci reaching genome-wide significance. Transcriptomic studies in samples with European ancestry nominate *ALCAM* and *BBX* as candidate protein-coding genes within the significant admixture mapping signal on 3q13.11, supported by the association between genetic variation and gene expression levels as well as differential expression between AD cases and controls. These results underline the power and challenges of leveraging genetic ancestry differences for new insight into the genetic architecture of late-onset AD in multiethnic populations.

## Methods

### Data

Genotype and phenotype data for 3067 participants in the Columbia University Study of Caribbean Hispanics and Late Onset Alzheimer’s Disease (CU Hispanics) were downloaded through dbGaP (Study Accession number phs000496.v1.p1), described in detail elsewhere [21]. The CU Hispanics study recruited subjects using both familial AD (22%) and sporadic case-control (78%) ascertainment. Subjects were excluded if they had any missing data for sex, AD status, *APOE* ε2/ ε3/ε4 genotypes, and either age-at-onset of dementia or age-at-last-evaluation.

European (from Utah) and African (Yorubans) samples from HapMap 3 [22] and Native American samples (Colombians, Pima, and Maya) from the Human Genome Diversity Project [23, 24] were used as reference populations. The reference datasets were merged using PLINK (v1.07) [25], resulting in 598,470 common autosomal single-nucleotide polymorphisms (SNPs). Genome coordinates were updated to build NCBI37/hg19 using LiftOver [26] to match the CU Hispanic data. The reference and CU Hispanics datasets were merged, randomly removing reference samples to balance ancestral population representation. Variants with a genotype missing rate > 5%, samples missing > 5% of genotypes, and 502 duplicated samples were excluded. Heterozygosity analysis identified 43 CU Hispanic outliers for both the F coefficient ( > 0.12, mean 0.02 ± 0.03) and heterozygosity rate (< 0.28, mean 0.32 ± 0.01), consistent with previous reports and pedigree documentation of consanguinity [27]. As both our admixture mapping and association tests adjust for genetic relatedness, keeping these samples had minimal impact on results (Additional file 1). The final combined dataset included 294,252 SNPs and 2754 samples: 2565 CU Hispanics plus 63 samples from each reference population. The overall genotyping rate was 0.993.

### Genetic relatedness matrix

A genetic relatedness matrix (GRM) was estimated in a recursive manner using the PC-AiR and PC-Relate functions within the GENESIS R package [28–30]. The final combined data set was included in these analyses to improve inference of population structure. PC-AiR partitions subjects into unrelated and related sets based on kinship estimates from KING-robust [31], performs principal components (PC) analysis on the set of unrelated subjects, then projects PC values for the related set. PC-Relate adjusted the GRM for the first four PCs derived by PC-AiR, and the PC-AiR and PC-Relate steps were repeated using this adjusted GRM. The final GRM contains kinship coefficients that are robust to the population structure within our sample.

### Ancestry proportions

As suggested by an established pipeline [32], the CU Hispanic and reference samples were phased jointly using ShapeIt2 [33] and 1000 Genomes phase 3 haplotypes [34] as reference. Local ancestry estimation was performed using RFMix (v1.5.4) [35]. Local ancestry values were averaged to estimate global European, African, and Native American ancestry proportions.

### Admixture mapping

Admixture mapping was performed using a logistic mixed model for the AD phenotype, in which all European, African, and Native American ancestries were tested simultaneously. Admixture mapping was conducted using the GENESIS R package [30] available in Bioconductor [36]. We fit mixed models under the null hypothesis of no genetic association, adjusting for global ancestry proportions and *APOE* ε2 and ε4 allele dosages as fixed effects and the GRM as a random effect. The association between each admixture linkage disequilibrium (LD) block and the null model was evaluated by a score test. Recent admixture, as such observed in Hispanic/Latinos, creates long-range LD which dramatically reduces the number of independent tests in an admixture mapping genome scan, leading to a less-severe multiple testing correction. Genome-wide significance was defined as *P* < 5E−05 and suggestive evidence for significance was defined as *P* < 0.001, as suggested by previous studies of Hispanic populations [37, 38]. We evaluated the suitability of these significance thresholds by extending the method proposed by Shriner et al. 2011 [39] for three ancestral populations. We estimated the effective number of tests for each ancestral population by fitting autoregressive models to the vectors of African, European, and Native American local ancestry dosages in our sample (African: 251.1, European: 210.3, Native American: 281.2) and defined the final effective number of tests as the sum of the two largest values. This Bonferroni-corrected significance threshold of *P* < 9.39E−05 is slightly less conservative than our original threshold, suggesting it is well-suited for this sample.

Secondary admixture mapping analyses considered the effect of each reference group separately to identify which ancestral population was driving the significant signals. The coefficients of each lead SNP in the most significant LD-block were estimated, taking the allelic dosage of the ancestry driving the signal into account. Manhattan plots were prepared using the qqman R package [40], while regional association plots were generated using LocusZoom [41]. Additional sensitivity analyses assessed the robustness of our findings to age and sex covariate adjustment.

### Association testing

SNPs and samples were submitted to the data cleaning procedures described above without the inclusion of reference samples, leaving 931,670 SNPs and 2565 CU Hispanic samples. We conducted the association testing for AD using a logistic mixed model implemented in the GENESIS R package [30]. Using the fitted null model described above, we tested the association between each SNP and the phenotype with a score test. Genome-wide significance was defined as *P* < 5E−08. Region-specific thresholds within the 3q13.11 locus for significant (*P* < 6.74E−05) and suggestive (*P* < 1.35E−05) evidence for association were adjusted for the effective number of tests, estimated by Genetic Type I error calculator [42].

### Locus interpretation and gene prioritization

Conditional admixture mapping analyses were performed, applying the original model with further adjustment for allele dosage at SNPs of interest, individually and jointly. LD was estimated by both r^2^ and D’ using PLINK [25] in a set of 1349 unrelated CU Hispanics. LD plots based on the correlation statistic D’ by reference population were prepared using Haploview [43]. The Ensembl Variant Effect Predictor (v99 [44]) toolset generated SNP-level annotations within regions of interest.

The Accelerating Medical Partnerships for Alzheimer’s Disease (AMP-AD) project has provided a publicly available repository of multi-omic data aimed at finding genetic targets for AD therapeutics. We extracted significant *cis* expression quantitative trait loci (*cis*-eQTLs) from a recent AMP-AD study [45] (https://www.synapse.org/#!Synapse:syn17015233), representing RNA-sequencing data generated on brain samples from the Mayo study, Religious Orders Study, Rush Memory and Aging studies, and Mount Sinai Brain Bank study generated across 7 tissues types: cerebellum (N = 261), temporal cortex (*N* = 262), dorsolateral prefrontal cortex (*N *= 573), inferior gyrus (*N* = 230), superior temporal gyrus (*N *= 225), frontal pole (*N* =260), and parahippocampal gyrus (*N *= 225). We extracted evidence for differential gene expression in post-mortem brain tissues between those affected by AD and controls from another AMP-AD study [46] (https://www.synapse.org/#!Synapse:syn11914606), restricted to the meta-analysis results from the random effects model. A false discovery rate (FDR) cutoff of < 0.05 provided by the AMP-AD studies was applied to both the differential gene expression and eQTL results.

The genome is organized into topologically associated domains (TADs) in three-dimensional space, where genes within the same TAD are more likely to be regulated by common *cis*-regulatory elements and transcription factors. Genes within the same TAD as the haplotypes associated with AD were extracted from the 3D Genome Browser [47] and the human dorsolateral prefrontal cortex data (DLPFC) [48], again using the study-specific FDR < 0.05 as the significance threshold.

Genetic variation and patterns of LD vary across populations, and ideally colocalization analyses should use association and eQTL results representing the same population; unfortunately, large eQTL studies of Caribbean Hispanic populations are unavailable. Colocalization analyses comparing our admixture mapping or association studies are restricted to comparisons with the AMP-AD eQTLs representing samples with primarily European ancestry, which may identify relationships between eQTLs and AD risk shared between these populations [49, 50]. Approximate Bayes factor colocalization was performed using the Coloc package in R (v3.2-1) [50], which computes five posterior probabilities: PP0 = no association with either trait; PP1 = association with trait 1 but not trait 2; PP2 = association with trait 2 but not trait 1; PP3 = association with both traits, two independent causal SNPs; and PP4 = association with trait 1 and trait 2, one causal SNP shared for both traits. The LocusCompareR package in R (v1.0.0) [51] illustrated the correlation between admixture mapping or association results and eQTL data.

## Results

### Sample description

**Fig. 1:**
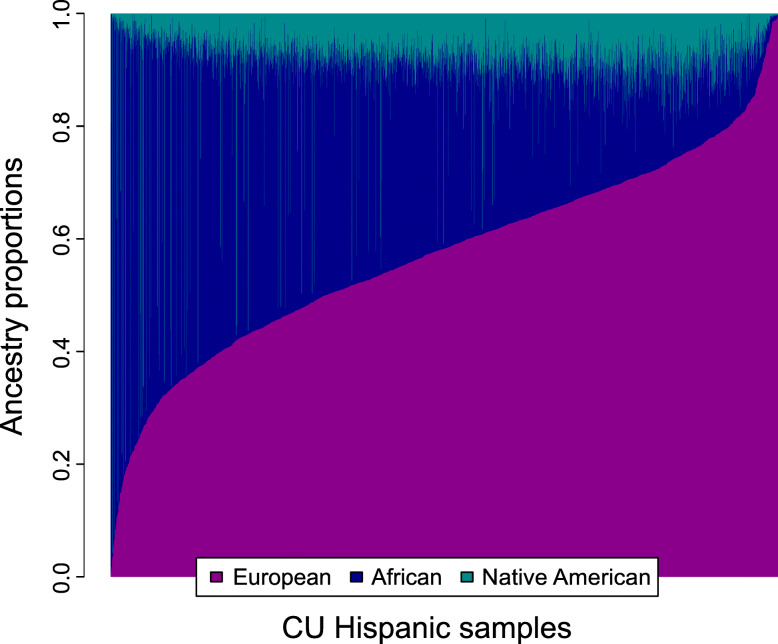
Estimated global ancestry proportions within the Caribbean Hispanics. X-axis: samples sorted by proportion of European ancestry, Y-axis: estimated global ancestry proportion. Colors correspond to reference populations: Blue for African, Purple for European, and Cyan for Native American

The CU Hispanics data represented 2565 subjects, where the 1174 cases were affected either by familial AD (22%) or sporadic AD (78%). Age-at-onset ranged from 44 to 100 years while the censoring age among the unaffected controls ranged from 35 to 100 years. The mean age and sex are similar across cases and controls (Table 1). The frequency of the protective *APOE* ε2 allele [52] is approximately 35% lower among cases, while the well-established risk allele ε4 [4, 5] is almost twice as common among cases than controls (Table 1). Global average ancestry proportion estimates vary widely across samples, from nearly zero to 0.99 per reference population (Fig. 1). Average ancestry proportions are 0.58 ± 0.17 European, 0.33 ± 0.19 African, and 0.09 ± 0.08 Native American ancestry.

**Table 1:** Sample description

Sample	*N*	Females (%)	Age (years)	ε2 (%)	ε4 (%)
Affected	1174	64.1	74.9 (9.4)	4.4	28.0
Unaffected	1391	67.7	72.5 (8.5)	6.7	14.5
Total	2565	66.1	73.6 (9.0)	5.6	20.7

Definitions: *N* = sample size, Age = mean ± standard deviation of age-at-onset of dementia (affected) or age-at-last-evaluation (unaffected), ε2 = frequency of the *APOE* ε2 allele, ε4 = frequency of the *APOE* ε4 allele

### Admixture mapping and GWAS

**Fig. 2:**
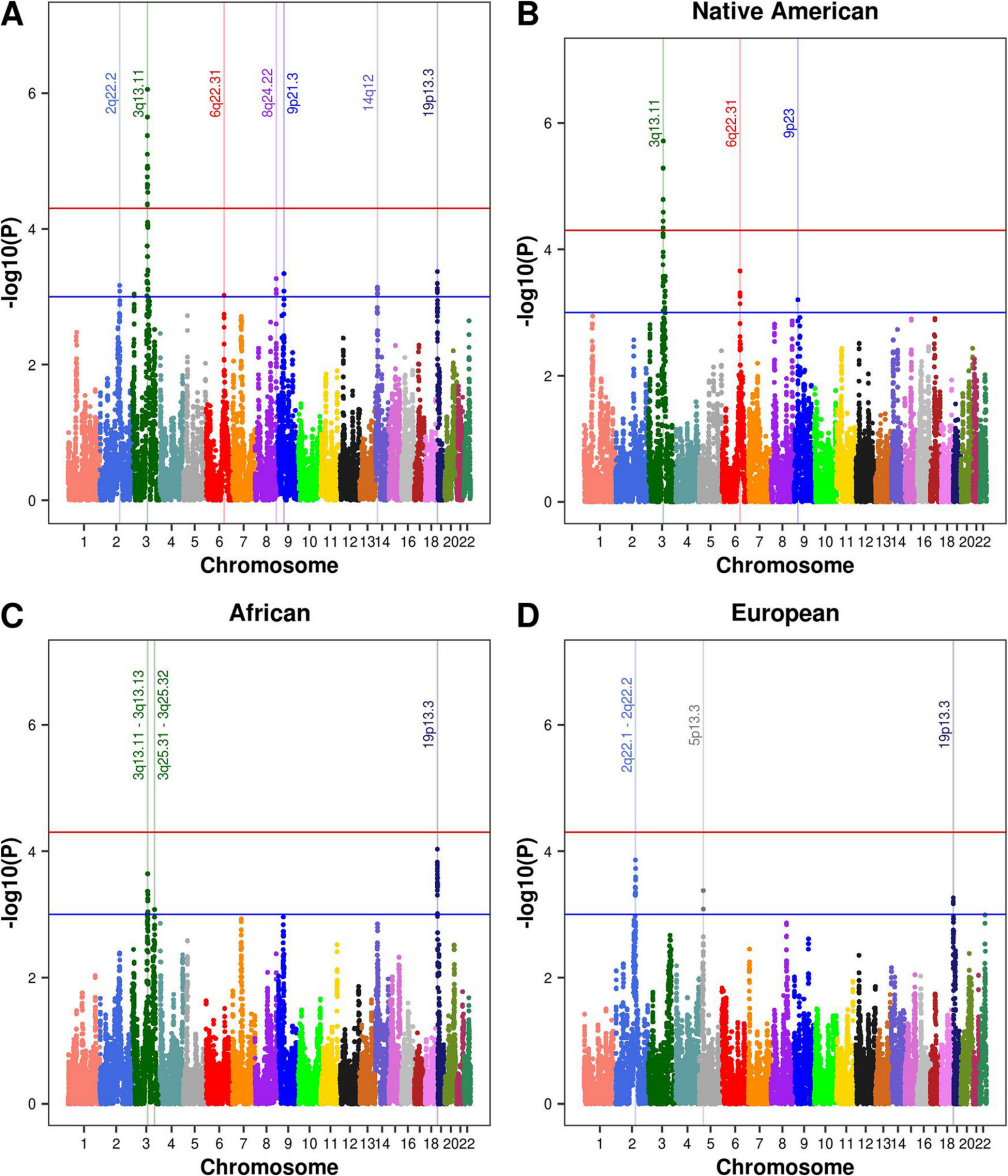
Association between Alzheimer’s disease and local ancestry among Caribbean Hispanics. Panel **a**: the joint European, African, and Native American ancestries admixture mapping analysis. Panels **b**, **c**, and **d**: results from single ancestry admixture mapping analyses for Native American, African, and European ancestries, respectively. Significant and suggestive thresholds represented by red and blue lines, respectively. Loci with significant or suggestive evidence of association with Alzheimer’s disease are highlighted with vertical bars labeled with their chromosomal position

We identified a genome-wide significant association between AD and local ancestry at 3q13.11 (*P* < 5E−05; Table 2, Fig. 2). The 3q13.11 signal is supported by significant evidence of association across multiple LD-blocks (103.7 to 107.7Mb, min. *P* = 8.76E-07; Table 2), where the lead SNP rs10933849 is a common variant across the ancestral populations (alternate frequency: 0.56, 0.84, and 0.61 for 1000 Genomes phase 3 Africans, Europeans, and Native Americans, respectively). This region spans five protein-coding genes: *ALCAM, CBLB, BBX, CCDC54,* and *CD47*. Secondary analyses indicated that Native American ancestry at the lead SNP of each LD-block was associated with a protective effect against AD risk (OR 0.58**–**0.66; *P* < 3.24E-04; Additional file 2). Greater correlation is observed between 15 SNPs tagging the LD blocks within the 3q13.11 locus in the Native American reference data than in the European or African data (Additional file 3), providing further evidence that the admixture mapping signal between AD and 3q13.11 is driven by a Native American haplotype.

**Table 2:** Evidence of association between local ancestry and Alzheimer’s disease in the Caribbean Hispanics

Chr	Position	rsID	Gene	Consequence	Ancestry	OR (95% CI)	***P***
2q22.2	142,486,253–143,387,612	rs13024316	*LRP1B*	Intron	EUR	1.28 (1.13−1.45)	6.82E−04
**3q13.11**	**103,747,624–107,725,831**	**rs10933849**	**Intergenic**	**Intergenic**	**NAM**	**0.58 (0.47−0.73)**	**8.76E−07**
6q22.31	123,548,997–123,838,033	rs6940177	*TRDN*	Intron	NAM	1.44 (1.19−1.75)	9.54E−04
8q24.22	135,308,849–135,856,404	rs4308771	RP11-513H8.1	Intron	NAM	1.36 (1.12−1.65)	5.41E−04
9p21.3	22,207,037–22,870,294	rs4977586	Intergenic	Intergenic	NAM	0.70 (0.56−0.87)	4.56E−04
14q12	32,485,703–33,033,695	rs1952961	RP11-187E13.2	Intron	AFR	0.81 (0.71−0.93)	7.24E−04
19p13.3	266,034–1,505,874	rs3787017	*PALM*	Intron	AFR	1.29 (1.14−1.47)	4.26E−04

Values are given for the SNP with the smallest *P* value per locus. Definitions: Chr = chromosome, Position = base pair position in NCBI37/hg19 genome build, Lead SNP = single-nucleotide polymorphism with the smallest P value within each LD-block, Ancestry = African (AFR), European (EUR), and Native American (NAM), OR = odds ratio, genome-wide significant evidence for association = *P* < 5E−05, suggestive evidence for association = *P* < 0.001. The block with significant evidence for association is highlighted in bold font. All intronic variants are canonical transcripts. Results for each block associated with Alzheimer’s disease at each locus are provided in Additional file 2

Suggestive evidence of association between local ancestry and AD was observed at six additional loci: 2q22.2, 6q22.31, 8q24.22, 9p21.3, 14q12, and 19p13.3 (*P* < 0.001; Fig. 2, Table 2). LD-block-specific results for significant and suggestive associations with AD are provided in Additional file 2. Two LD-blocks with European background were responsible for the suggestive signal at 2q22.2, intersecting the gene *LRP1B*. The suggestive signal on 6q22.31 is driven by the Native American ancestry and was captured by a single LD-block within the *TRDN* gene. On 8q24.22, we observed three LD-blocks with Native American background driving the signal which spans the *ZFAT* gene. Two LD-blocks spanning the *DMRTA1* gene were responsible for the signal on 9p21.3, driven by the Native American ancestry. The signal on 14q12, driven by the African ancestry, was captured by five LD-blocks implicating *ARHGAP5* and *AKAP6*. Nine LD-blocks within a 1.3Mb region were responsible for the signal on 19p13.3 driven by African ancestry, implicating *ABCA7* and dozens of other genes (Table 2). Sensitivity analyses revealed the admixture mapping results are robust to the inclusion of age and sex as covariates (Additional file 4). In contrast, traditional GWAS for AD did not identify any loci reaching genome-wide significance (*P* < 5E-08; Additional file 5).

### Locus interpretation and gene prioritization

**Fig. 3:**
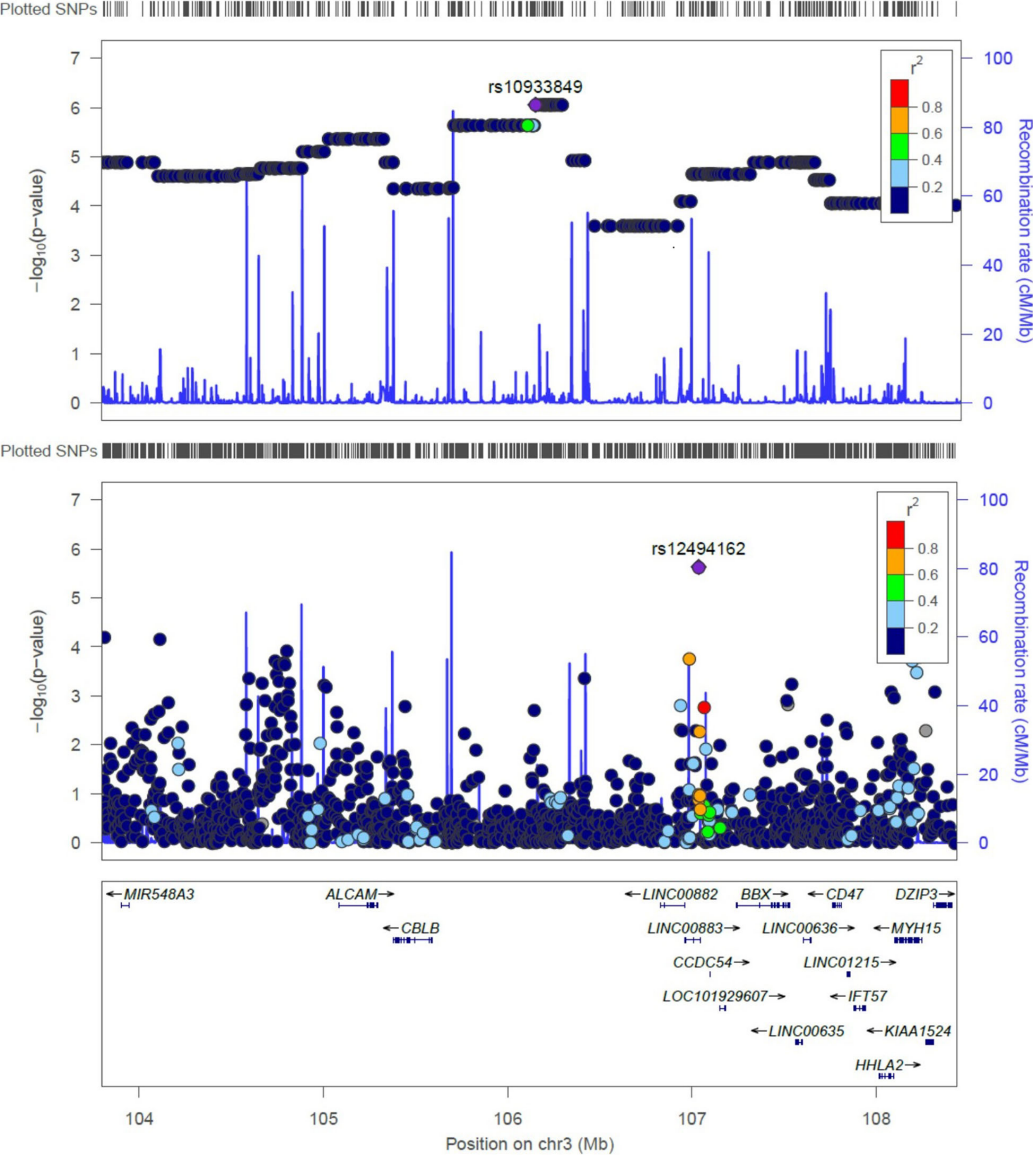
Patterns of linkage disequilibrium within the admixture mapping (top) and association (bottom) signals at 3q13.11. LocusZoom plots were drawn using the 1000 Genomes Native American estimates of linkage disequilibrium (r^2^; Nov. 2014). Chromosomal position on the hg19 map is shown on the X-axis, while the Y-axis provides evidence of association with Alzheimer’s disease as the –log_10_(*P*) value

Targeted association testing within the 3q13.11 locus found two SNPs significantly associated with AD (rs12494162, *P* = 2.33E-06; rs1731642, *P* = 6.36E-05), and 22 SNPs with suggestive evidence of association with AD (*P* < 1.35E-03; Table 3). The first SNP, rs12494162, falls within an intron of lncRNA *DUBR*, while rs1731642 is an intergenic variant. These two SNPs, rs12494162 and rs1731642, are not in LD within our data (r^2^ = 0.003; D’= 0.17) and may represent independent association signals. This is consistent with LocusZoom plots of the admixture mapping and association signals at 3q13.11 using 1000 Genomes Native American estimates of LD (Fig. 3). The lead SNP rs12494162 is in LD with several other SNPs with evidence of association with AD, as expected. In contrast, the lead SNP from the admixture mapping analysis has modest evidence of LD with other SNPs on haplotypes associated with AD, as the admixture mapping signal is driven by differences in ancestry proportions rather than specific genotypes at the locus.

**Table 3:** Variants with significant or suggestive evidence for association with Alzheimer’s disease within 3q13.11

Chr	SNP	Position	Allele	Gene	Consequence	OR (95% CI)	***P***
**3**	**rs1731642**	**103,811,750**	**G**	**38kb 5' of AC016970.1**	**intergenic**	**1.33 (1.17—1.53)**	**6.36E**−**05**
3	rs9848147	104,111,719	C	338kb 5' of AC016970.1	intergenic	1.65 (1.30**—**2.08)	7.02E−05
3	rs2673478	104,158,244	G	385kb 5' of AC016970.1	intergenic	1.22 (1.09**—**1.38)	1.33E−03
3	rs16850638	104,594,467	A	491kb 5' of *ALCAM*	intergenic	0.69 (0.57**—**0.84)	4.24E−04
3	rs12492893	104,667,061	A	419kb 5' of *ALCAM*	intergenic	1.23 (1.10**—**1.38)	5.45E−04
3	rs9288795	104,683,753	G	402kb 5' of *ALCAM*	intergenic	0.83 (0.74**—**0.92)	1.21E−03
3	rs16850772	104,736,837	A	349kb 5' of *ALCAM*	intergenic	1.26 (1.12**—**1.41)	1.91E−04
3	rs9883825	104,745,091	G	341kb 5' of *ALCAM*	intergenic	0.80 (0.71**—**0.90)	3.55E−04
3	rs13325696	104,755,481	A	330kb 5' of *ALCAM*	intergenic	1.28 (1.13**—**1.45)	2.29E−04
3	rs1503079	104,759,032	A	327kb 5' of *ALCAM*	intergenic	0.81 (0.72**—**0.91)	4.92E−04
3	rs1566720	104,761,408	A	324kb 5' of *ALCAM*	intergenic	0.82 (0.73**—**0.92)	1.13E−03
3	rs1587707	104,786,133	C	300kb 5' of *ALCAM*	intergenic	0.80 (0.71**—**0.89)	2.06E−04
3	rs2895295	104,790,415	C	295kb 5' of *ALCAM*	intergenic	0.80 (0.71**—**0.90)	2.32E−04
3	rs10933809	104,800,350	A	285kb 5' of *ALCAM*	intergenic	0.79 (0.70**—**0.89)	1.19E−04
3	rs1503089	104,805,417	G	280kb 5' of *ALCAM*	intergenic	1.23 (1.09**—**1.39)	9.28E−04
3	rs1503075	104,806,853	G	279kb 5' of *ALCAM*	intergenic	0.81 (0.72**—**0.92)	1.23E−03
3	rs1503158	104,815,105	G	271kb 5' of *ALCAM*	intergenic	0.80 (0.71**—**0.90)	5.52E−04
3	rs13322578	105,002,637	A	83kb 5' of *ALCAM*	intergenic	0.77 (0.66**—**0.89)	5.90E−04
3	rs9816851	105,012,805	G	73kb 5' of *ALCAM*	intergenic	0.77 (0.67**—**0.89)	6.64E−04
3	rs9860520	106,416,700	A	9.1kb 3' of Y_RNA	intergenic	0.79 (0.70**—**0.90)	4.28E−4
3	rs12489299	106,985,680	A	*DUBR*	intron	0.69 (0.57**—**0.83)	1.70E−04
**3**	**rs12494162**	**107,036,379**	**A**	***DUBR***	**Intron**	**0.69 (0.60—0.80)**	**2.33E**−**06**
3	rs7615167	107,514,134	A	*BBX*	Intron	1.56 (1.20**—**2.02)	1.23E−03
3	rs4855772	107,540,375	G	10kb 3' of BBX	intergenic	1.80 (1.31**—**2.49)	5.81E−04

Significance thresholds are based on the effective number of independent tests. Abbreviations: Chr = chromosome, SNP = single-nucleotide polymorphism identifier, Position = chromosome 3 position on the GRCh37/hg19 map, Allele = effect allele, OR = odds ratio, significant evidence of association = *P* < 6.7E**-**05, suggestive evidence of association = *P* < 1.4E**-**03. Significant results are highlighted in bold font

**Fig. 4:**
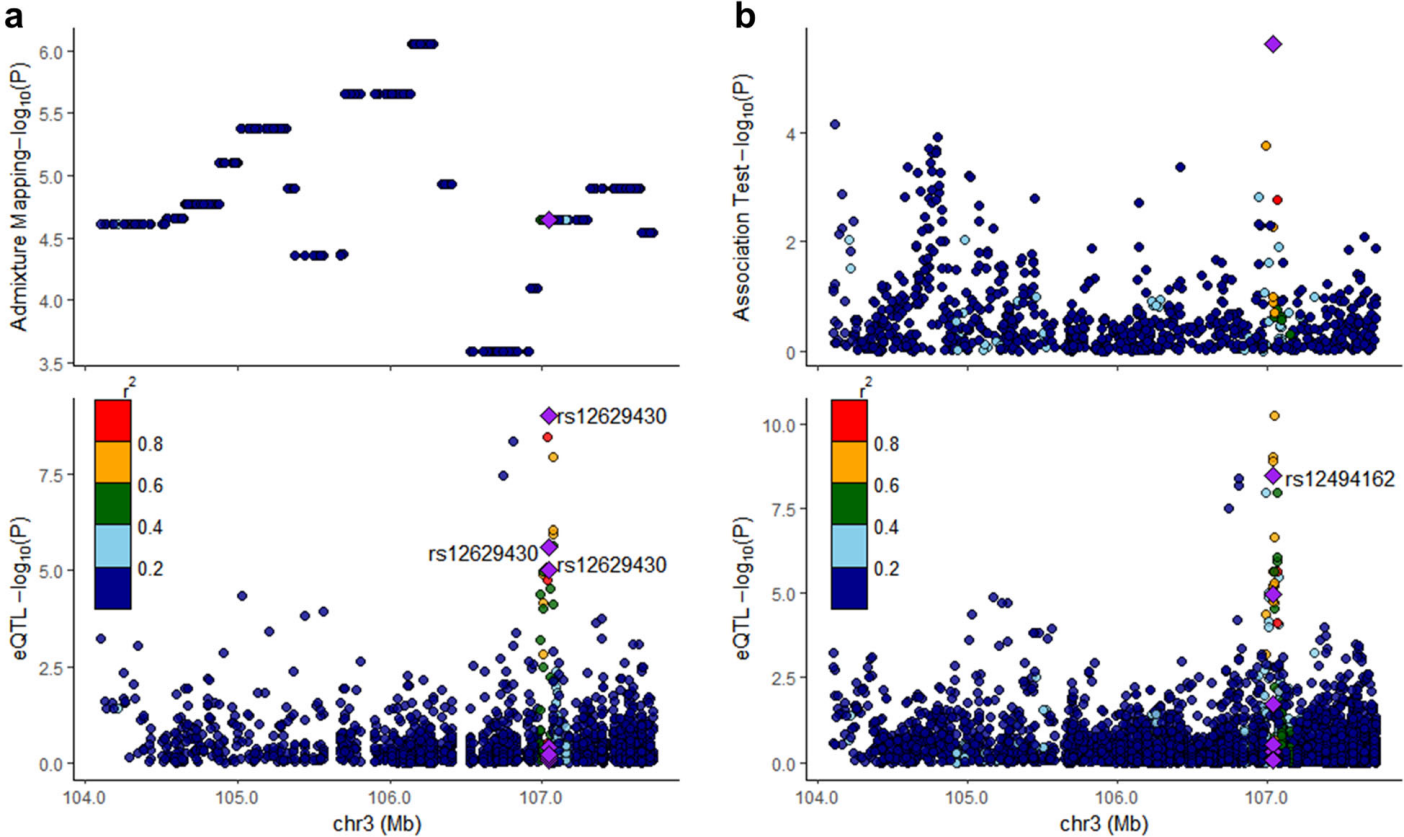
Colocalization between *cis*-eQTLs and admixture mapping (**a**) and association results (**b**) at 3q13.11. **a** Colocalization results between significant admixture mapping haplotypes and eQTLs from dorsolateral prefrontal cortex (DLPFC) data. The color scale depicts extent of linkage disequilibrium (LD) with the lead *cis*-eQTL (rs12629430, purple diamond) in the 1000 Genomes Native American sample, which falls within a significant haplotype block. **b** Colocalization results between association test of 3q13.11 and *cis*-eQTLs from DLPFC data. The color scale depicts the amount of LD with the lead SNP from the association tests (rs12494162, purple diamond), based on 1000 Genomes Native American samples. The lead SNP rs1249162 is a significant *cis-*eQTL in the DLPFC data. Note: the difference in eQTL plots between panels **a** and **b** are due to differences in SNP marker density between admixture mapping and association testing

Conditional admixture mapping analyses including both rs12494162 and rs1731642 as covariates eliminated the signal at 3q13.11 (*P* = 0.01), while analyses conditioned on either SNP alone only weakened the signals (Additional file 6), suggesting that admixture mapping and GWAS approaches may be tagging the same underlying variant. We assessed evidence of colocalization between eQTLs identified in DLPFC samples from subjects with primarily European ancestry and the admixture mapping and association signals at 3q13.11, as comparable studies representing Native Americans are unavailable. These analyses can only identify shared genetic architecture between eQTLs shared across populations and our admixture mapping or association results, which may represent fewer than half of eQTLs [49]. The leading eQTL within the 3q13.11 locus falls within a haplotype significantly associated with AD in the admixture mapping analysis (Fig. 4a): rs12629430 is significantly associated with the expression of lncRNA *DUBR* (Z = −4.47, FDR = 4.9E−04), lincRNA RP11.446H18.1 (Z = −6.22, FDR = 1.0E−07), and lincRNA RP11-446H18.6 (Z = −4.77, FDR = 1.4E−04). Colocalization analyses of the eQTL and admixture mapping signals did not reject the null hypothesis (PP0 = 0.9550). In contrast, the lead SNP from our targeted association testing within 3q13.11, rs12494162, is also an eQTL significantly associated with the expression of lincRNA RP11.446H18.1 (Z =−6.01, FDR = 3.4E−07), and lincRNA RP11-446H18.6 (Z =−4.44, FDR = 5.4E−04) (Fig. 4b). Colocalization analyses are not significant, but suggest association with both AD and gene expression here and weakly favor the model of independent SNPs driving these association (PP3 = 0.5070) rather than one shared SNP (PP4 = 0.4130).

Within the 3q13.11 locus (chr3:103,747,624-107,725,831), we prioritized candidate protein-coding genes which fell either within one of the 15 LD-blocks associated with AD or within an intersecting TAD using the following features in transcriptomic studies representing European ancestry: (1) genes in which expression in brain tissue is significantly associated with *cis*-eQTL within the region of interest and (2) genes which are differentially expressed in the brain between AD cases and controls. The 3q13.11 region of interest spans five protein-coding genes: *ALCAM, BBX, CBLB, CCDC54,* and *CD47* while four additional genes fall within the same TAD as *BBX*: *IFT57, HHLA2*, *MYH15*, and *KIAA1524* (Additional file 7). A recent transcriptomic study [45] of 1694 brain samples identified 369 significant *cis*-eQTLs for *IFT57*, 182 significant *cis*-eQTLs for *ALCAM*, 118 significant *cis*-eQTLs for *CBLB*, 47 significant *cis*-eQTLs for *CD47*, 22 significant *cis*-eQTLs for *BBX*, and 6 significant *cis*-eQTLs for *MYH15* (FDR < 0.05). The strongest *cis*-eQTL per gene is reported in Table 4, with all *cis*-eQTLs reported in Additional file 8. Another transcriptomic study [46] including an overlapping sample set of 2114 brain samples representing 478 cases and 300 controls identified significant evidence for differential gene expression in AD for both *ALCAM* (Z = 2.75, FDR = 2.76E-02) and *BBX* (Z = 3.73, FDR = 1.84E-03) (Additional file 9). While variation in the 3q13.11 region is associated with expression levels of *ALCAM*, *BBX*, *CBLB*, *CD47 IFT57*, and *MYH15* in the brain, only *ALCAM* and *BBX* were significantly differentially expressed between AD cases and controls.

**Table 4:** Significant *cis* expression quantitative loci (eQTLs) for candidate genes within 3q13.11 region of interest

Position	SNP	Gene	Z	β	Allele	***P***	FDR
105,108,867	rs6797043	*ALCAM*	**-**4.89	**-**0.3429	C	1.29E**-**06	8.24E**-**05
107,583,197	rs9875001	*BBX*	3.72	0.2620	T	2.17E**-**04	7.43E**-**03
105,488,134	rs139969708	*CBLB*	4.01	0.4411	T	7.03E**-**05	2.85E**-**03
107,550,819	rs1908324	*CD47*	3.83	0.2512	T	1.45E**-**04	5.29E**-**03
107,917,824	rs9857584	*IFT57*	6.91	0.6714	C	1.33E**-**11	1.89E**-**09
107,823,224	rs80003826	*MYH15*	**-**3.92	**-**0.4498	A	9.85E**-**05	3.80E**-**03

Results are filtered to only include those with false discovery rate (FDR) < 0.05, restricted to the SNP with the smallest FDR value. Results for all significant eQTLs are presented in Additional file 8 and the original data can be found at https://www.synapse.org/#!Synapse:syn17015233. Legend: Position = GRCh37 position of the variant on chromosome 3, Z = Z statistic, β = estimated effect size, allele = effect allele, FDR = false discovery rate

## Discussion

Admixture mapping of AD within a Caribbean Hispanic sample identified one genome-wide significant signal on 3q13.11 (*P* = 8.76E-07) and six unique suggestive signals at 2q22.2, 6q22.31, 8q24.22, 9p21.3, 14q12, and 19p13.3. The admixture mapping signal on 3q13.11 spanned 15 haplotype blocks, where the Native American ancestry is associated with reduced risk of AD. Association between the Native American ancestry and reduced risk of AD has previously been reported [53, 54]. Suggestive evidence of association between the 3q13.11 locus and AD has recently been reported in an African American GWAS involving nearly three times the sample size as our study [55], demonstrating the effectiveness of the admixture mapping approach as a complement to GWAS.

While admixture mapping provides insights into the genetic basis of disease in multiethnic populations, integration of AD transcriptomics allowed us to nominate candidate genes within 3q13.11. *ALCAM* and *BBX*, the genes with significant evidence for both brain eQTLs and differential expression between AD cases and controls, both have robust support in the literature for a functional relationship to AD. Proteomic studies suggest *ALCAM*, which plays a role in neuron-neuron adhesion and neurite growth networks, is dysregulated during the progression of AD [56]. *ALCAM* is also involved in blood-brain barrier disruption and T cell-dependent neurodegeneration [57], biological pathways implicated in the progression of AD [58]. Furthermore, *ALCAM* is a target gene of miR-142 which is significantly upregulated in the AD brain [59, 60]. *BBX* is differentially expressed in the entorhinal cortex and hippocampus and appears to play a role in the crosstalk between the peripheral blood and the central nervous system [61]. Multiple studies have shown that *BBX* is differentially expressed in the AD brain [61, 62], while another implicated *BBX* as a candidate Master Regulator responsible for AD progression [63].

Each of the loci harboring suggestive admixture mapping signals have also been previously associated with AD risk and/or pathology. The signal on 19p13.3 is driven by African ancestry and spans *ABCA7*, a gene in which coding changes have been associated with risk of AD in both African American and European American samples [64–67]. *LRP1B* within the 2q22.2 signal has been implicated in the production and presentation of amyloid beta (Aβ) [68], while multiple *LRP1B* haplotypes are associated with risk of developing AD in studies representing European Americans [69] and Caribbean Hispanics [70]. Variants on 14q13.1 near *NPAS3* have been associated with AD biomarkers [71] and general cognitive function [72]. Variants in *ZFAT* on 8q24.22 have been associated with extreme longevity [73] and cerebrospinal fluid *tau/Aβ42* levels [74]. Within 6q22.31, *TRDN* variants have implicated in cerebral Aβ deposition in *APOE* ε4 non-carriers [75] and rate of cognitive decline in AD [76]. Finally, 9p21.3 has previously been linked to AD risk [77], and variants within the region have been associated with both vascular dementia and AD [78].

### Limitations

Our study has several limitations. Admixture mapping identifies regions associated with a given trait which must then be fine-mapped to identify the underlying risk variants. Colocalization analysis is not well powered in our study due to the poor representation of non-European populations in large eQTL data sets, as the genetic architecture of eQTLs can be ancestry specific [49, 79]. Fine-mapping analyses of whole-genome sequence data collected in this sample may allow the detection of the variants responsible for the admixture mapping signals. Publicly available datasets comparable in size or Native American ancestry proportions suitable for replication analyses are not available. Ongoing efforts, including AMP-AD and the Alzheimer’s Disease Sequencing Project, will provide data which may assist these efforts in the future.

## Conclusions

Most AD GWAS have represented samples with European ancestry, and alternative strategies may detect additional genetic variants influencing AD in multiethnic populations. Caribbean Hispanics, despite being more likely to be diagnosed with AD [80, 81], have been underrepresented in AD genetics studies [82]. We illustrated the power of admixture mapping for detecting loci associated with AD in a Caribbean Hispanic sample, provided robust evidence for this association, and nominated several candidate genes with orthogonal functional and statistical evidence for a role in AD. Further investigation of these loci and nominated genes could lead to a better understanding of the genetic heterogeneity of AD in populations with significant Native American ancestry.

## Supplementary Information

**Additional file 1: **Joint Photographic Group format .jpg file. Admixture mapping of Alzheimer’s disease in the Caribbean Hispanics excluding the heterozygosity outlier samples. Description: Joint European, African and Native American ancestries admixture mapping analysis, with chromosomal position on hg19 on the X-axis and –log10(*P*) values on the Y-axis. Significant and suggestive thresholds represented by red and blue lines, respectively. Loci with significant or suggestive evidence of association with Alzheimer’s disease are highlighted with vertical bars labeled with the chromosomal position of the peak.

**Additional file 2:** File format: Microsoft Word .docx file. Title: Linkage disequilibrium blocks within regions with either genome-wide significant or suggestive evidence for association between local ancestry and Alzheimer’s disease. Description: Regions reaching at least suggestive evidence of association with Alzheimer’s disease are defined and the evidence for that association is summarized. Abbreviations: Chr = chromosome, Position = physical positions based on GRCh37/hg19 map, SNP = single nucleotide polymorphism, Effect size (95%CI): odds ratio for AD followed by the 95% confidence interval.

**Additional file 3:** File format: Portable graphics format .png file. Title: Linkage disequilibrium patterns at 3q13.11 by ancestry. Description: Each panel illustrates the amount of linkage disequilibrium (D’) between pairs of markers in the 3q13.11 locus using different reference populations drawn from the 1000 Genomes data (Nov 2014).

**Additional file 4: **File format: Joint Photographic Group format .jpg file. Title: Admixture mapping of Alzheimer’s disease in the Caribbean Hispanics adjusted for age and sex. Description: Joint European, African and Native American ancestries admixture mapping analysis, with chromosomal position on hg19 on the X-axis and –log10(*P*) values on the Y-axis. Significant and suggestive thresholds represented by red and blue lines, respectively. Loci with significant or suggestive evidence of association with Alzheimer’s disease are highlighted with vertical bars labeled with the chromosomal position of the peak.

**Additional file 5: **File format: Portable graphics format .png file. Title: Genome-wide association testing results for Alzheimer’s disease. Description: Alzheimer’s disease status was tested for association with genotypes using a logistic regression model, adjusting for global ancestry proportions and *APOE* ε2 and ε4 allele dosages as fixed effects and the genetic relatedness matrix as a random effect. Genomic position on the hg19 map are provided on the X-axis and –log10(*P*) values on the Y-axis. The dotted horizontal line corresponds to a genome-wide significance threshold of 5E-08.

**Additional file 6: **File format: Portable graphics format .png file. Title: Conditional admixture mapping results at 3q13.11. Description: Each panel represents an admixture mapping analysis in the 3q13.11 locus, conditioned on the two single nucleotide polymorphisms (SNPs) associated with Alzheimer’s disease. The first panel shows the admixture mapping results with both SNPs are included in the analysis model, while the latter two adjust for only the named SNP. The X-axis represents the genomic position on chromosome 3 and the Y-axis represents –log10(*P*) values. The horizontal red line represents region-wide significance, while the blue line represents suggestive evidence of association. Green dots represent the locus reaching genome-wide significance in the original admixture mapping analysis.

**Additional file 7: **File format: Portable graphics format .png file. Title: Local context of admixture mapping and association signals at 3q13.11. Description: The top panel illustrates the admixture mapping testing for association with Alzheimer's disease status model. The second panel provides the association testing results for AD. The third panel provides the position of genes within the region of interest. The fourth panel illustrates the topologically associated domains (TADs; blue and gold bars) indicated by Hi-C experiments in DLPFC, where the heat map in magenta indicates the number of sequencing reads aligning to a pair of physical positions. Red horizontal lines represent the genome-wide significance threshold (*P* < 5E-05) and a blue line at the suggestive threshold (*P* < 0.001) used for admixture mapping. Sequence positions are aligned to the GRCh37/hg19 genome reference and are represented by the X-axis.

**Additional file 8: **File format: Microsoft Word .docx file. Title: Significant *cis* expression quantitative loci (eQTLs) for candidate genes within 3q13.11 region of interest. Description: Evidence that genotypes at a marker are associated with gene expression values. Results are filtered to only include those with false discovery rate < 0.05. Data are stored in the Synapse repository, Synapse ID: syn17015233, https://www.synapse.org/#!Synapse:syn17015233. Legend: Chr = chromosome, Position = GRCh37 position of the variant on chromosome 3, Z = Z statistic, FDR = false discovery rate, β = estimated effect size, A1 = allele 1, A2 = allele 2, A2freq = observed frequency of the A2 allele, Aup = allele associated with increased expression of the gene.

**Additional file 9: **File format: Microsoft Word .docx file. Title: Evidence of differential gene expression between those with and without Alzheimer's disease for 3q13.11 candidate genes. Description: Data are stored in the Synapse repository, Synapse ID: syn11914606, https://www.synapse.org/#!Synapse:syn11914606, file meta.anlz.ad_cntrl.tsv, which included no values for *CCDC54* or *HHLA2*. Legend: z.fixed = Z statistic for the fixed effects model, p.fixed = p-value for fixed effect model, z.random = Z statistic for the random effects model, p.random = p-value for the random effects model, fdr.fixed = false discovery rate from the fixed effects model, fdr.random = false discovery rate from random effects model. Results reaching the significance level of fdr.random < 0.05 are highlighted in bold.

## Data Availability

The genome scan data representing the Caribbean Hispanics are available through an application to the database of Genotypes and phenotypes (dbGaP; study ID: phs000496.v1.p1). These data are consented for general research use and are available through an application to dbGaP (https://www.ncbi.nlm.nih.gov/projects/gap/cgi-bin/study.cgi?study_id=phs000496.v1.p1). Analysis pipelines were based upon the GENESIS package in the R programming language (https://bioconductor.org/packages/release/bioc/html/GENESIS.html) and a published ancestry inference pipeline (https://github.com/armartin/ancestry_pipeline).

## Abbreviations

Aβ: Amyloid beta
AD: Alzheimer’s disease
AMP-AD: Accelerating Medical Partnerships for Alzheimer’s Disease
CU Hispanics: Participants in the Columbia University Study of Caribbean Hispanics and Late Onset Alzheimer’s Disease study
DLPFC: Dorsolateral prefrontal cortex
eQTL: Expression quantitative trait locus
FDR: False discovery rate
GRM: Genetic relatedness matrix
GWAS: Genome-wide association study
LD: Linkage disequilibrium
PC: Principal components
SNP: Single-nucleotide polymorphism
TAD: Topologically associated domain
