# Supplementary figures and images for: Admixture mapping reveals the association between Native American ancestry at 3q13.11 and reduced risk of Alzheimer’s disease in Caribbean Hispanics
Source: Alzheimers Res Ther. 2021 Jul 3;13:122. doi: 10.1186/s13195-021-00866-9 (PMC8254995; doi:10.1186/s13195-021-00866-9)

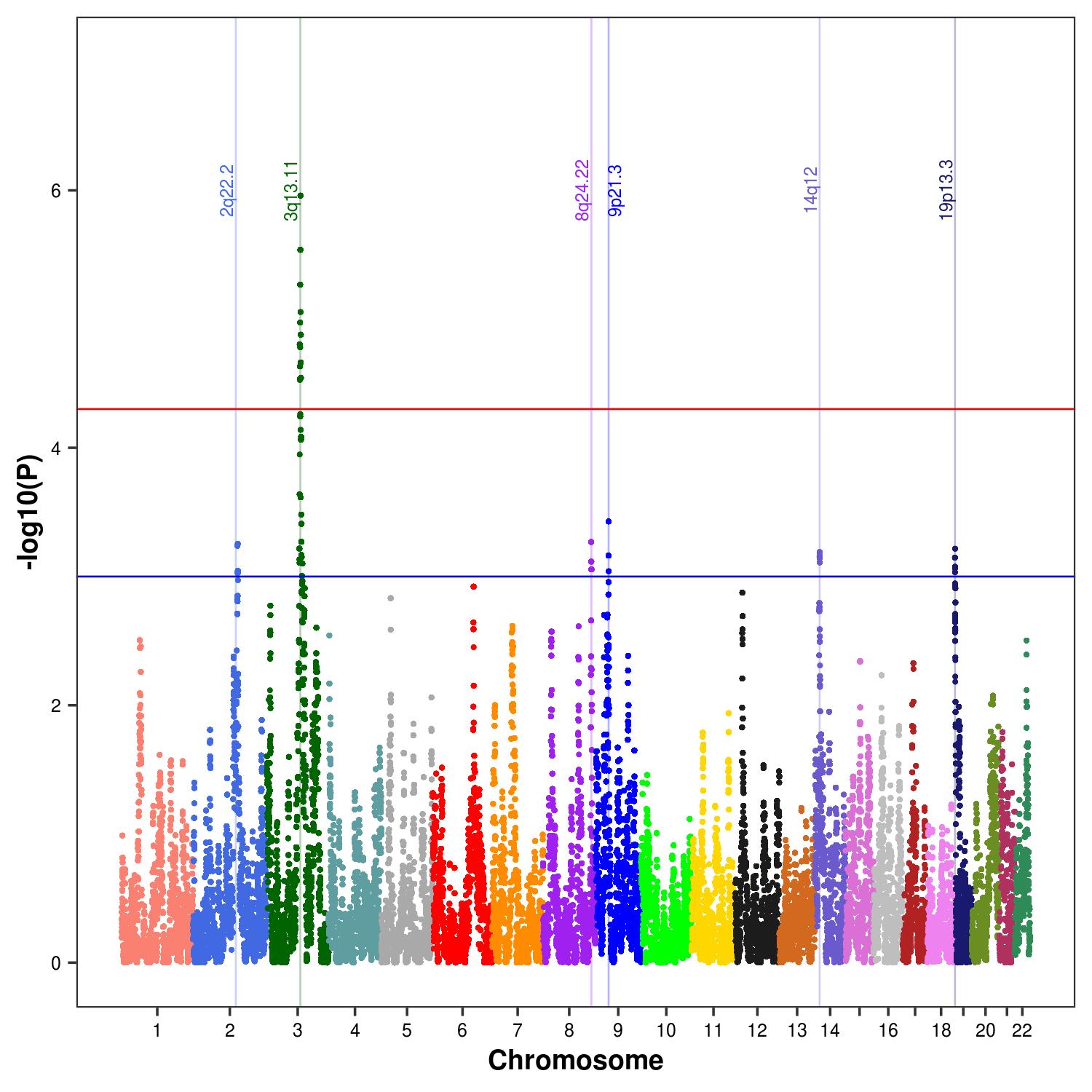

Supplement: Supplementary file 1 — Additional file 1: Joint Photographic Group format .jpg file. Admixture mapping of Alzheimer’s disease in the Caribbean Hispanics excluding the heterozygosity outlier samples. Description: Joint European, African and Native American ancestries admixture mapping analysis, with chromosomal position on hg19 on the X-axis and –log10(P) values on the Y-axis. Significant and suggestive thresholds represented by red and blue lines, respectively. Loci with significant or suggestive evidence of association with Alzheimer’s disease are highlighted with vertical bars labeled with the chromosomal position of the peak. [file 13195_2021_866_MOESM1_ESM.jpg]

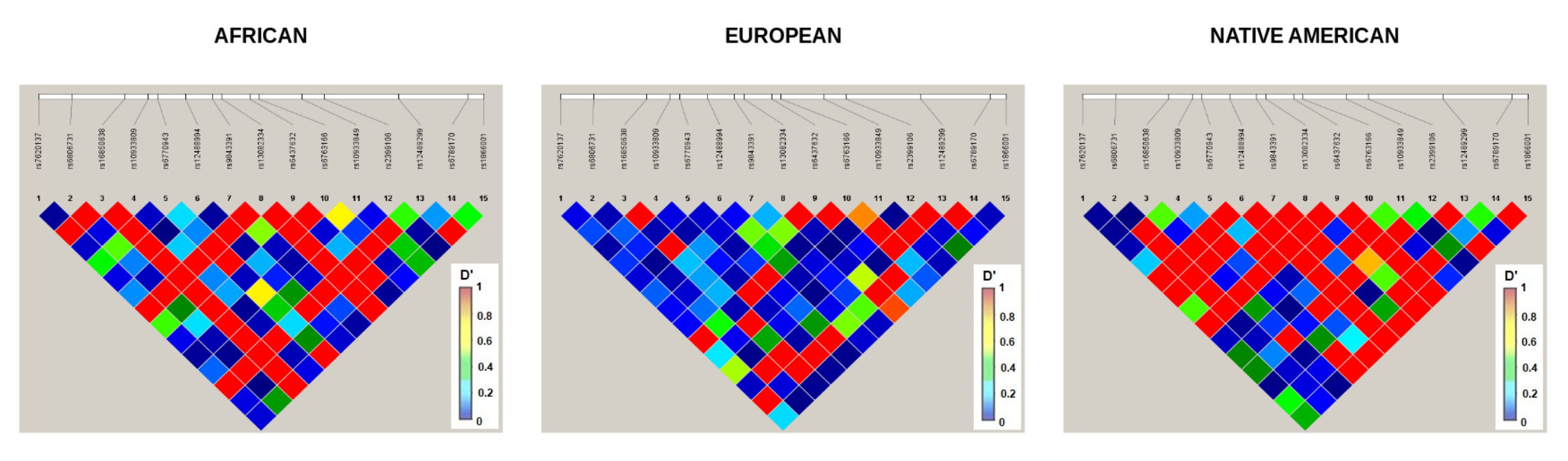

Supplement: Supplementary file 3 — Additional file 3: File format: Portable graphics format .png file. Title: Linkage disequilibrium patterns at 3q13.11 by ancestry. Description: Each panel illustrates the amount of linkage disequilibrium (D’) between pairs of markers in the 3q13.11 locus using different reference populations drawn from the 1000 Genomes data (Nov 2014). [file 13195_2021_866_MOESM3_ESM.png]

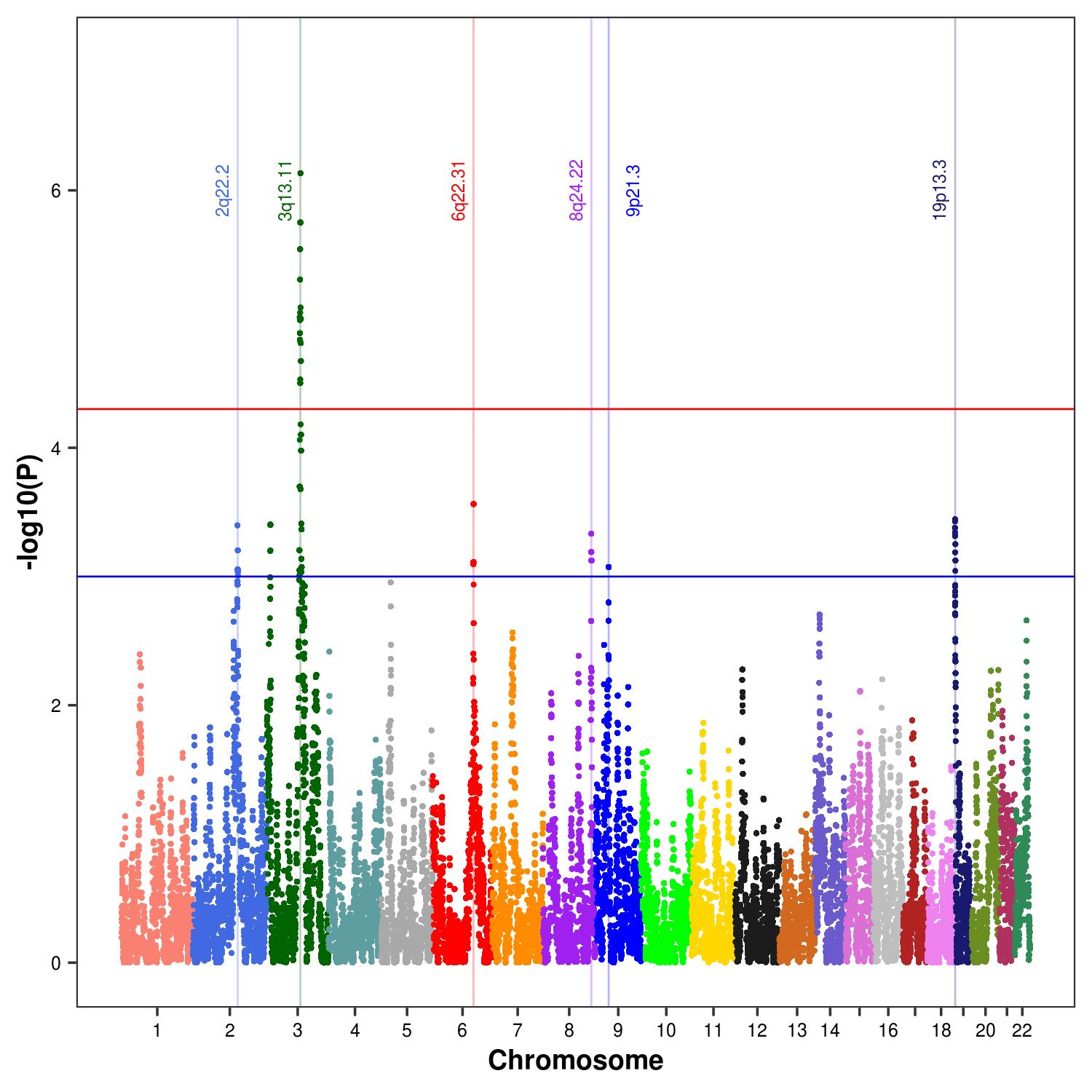

Supplement: Supplementary file 4 — Additional file 4: File format: Joint Photographic Group format .jpg file. Title: Admixture mapping of Alzheimer’s disease in the Caribbean Hispanics adjusted for age and sex. Description: Joint European, African and Native American ancestries admixture mapping analysis, with chromosomal position on hg19 on the X-axis and –log10(P) values on the Y-axis. Significant and suggestive thresholds represented by red and blue lines, respectively. Loci with significant or suggestive evidence of association with Alzheimer’s disease are highlighted with vertical bars labeled with the chromosomal position of the peak. [file 13195_2021_866_MOESM4_ESM.jpg]

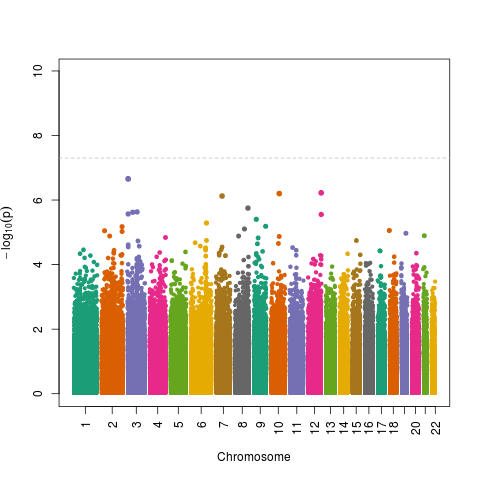

Supplement: Supplementary file 5 — Additional file 5: File format: Portable graphics format .png file. Title: Genome-wide association testing results for Alzheimer’s disease. Description: Alzheimer’s disease status was tested for association with genotypes using a logistic regression model, adjusting for global ancestry proportions and APOE ε2 and ε4 allele dosages as fixed effects and the genetic relatedness matrix as a random effect. Genomic position on the hg19 map are provided on the X-axis and –log10(P) values on the Y-axis. The dotted horizontal line corresponds to a genome-wide significance threshold of 5E-08. [file 13195_2021_866_MOESM5_ESM.png]

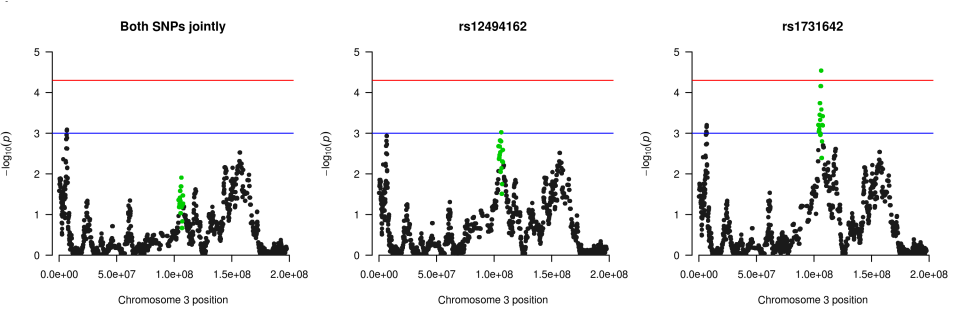

Supplement: Supplementary file 6 — Additional file 6: File format: Portable graphics format .png file. Title: Conditional admixture mapping results at 3q13.11. Description: Each panel represents an admixture mapping analysis in the 3q13.11 locus, conditioned on the two single nucleotide polymorphisms (SNPs) associated with Alzheimer’s disease. The first panel shows the admixture mapping results with both SNPs are included in the analysis model, while the latter two adjust for only the named SNP. The X-axis represents the genomic position on chromosome 3 and the Y-axis represents –log10(P) values. The horizontal red line represents region-wide significance, while the blue line represents suggestive evidence of association. Green dots represent the locus reaching genome-wide significance in the original admixture mapping analysis. [file 13195_2021_866_MOESM6_ESM.png]

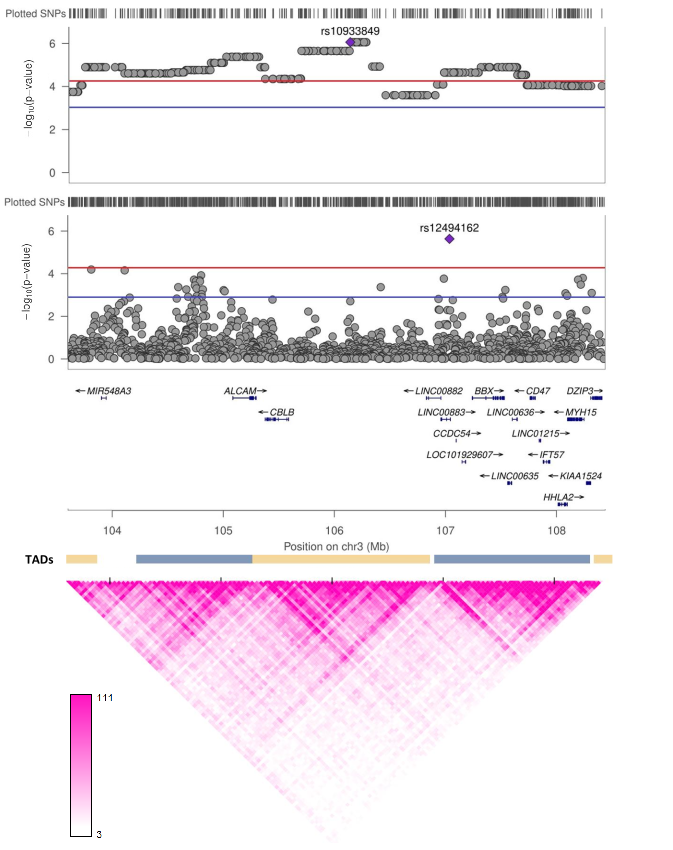

Supplement: Supplementary file 7 — Additional file 7: File format: Portable graphics format .png file. Title: Local context of admixture mapping and association signals at 3q13.11. Description: The top panel illustrates the admixture mapping testing for association with Alzheimer's disease status model. The second panel provides the association testing results for AD. The third panel provides the position of genes within the region of interest. The fourth panel illustrates the topologically associated domains (TADs; blue and gold bars) indicated by Hi-C experiments in DLPFC, where the heat map in magenta indicates the number of sequencing reads aligning to a pair of physical positions. Red horizontal lines represent the genome-wide significance threshold (P < 5E-05) and a blue line at the suggestive threshold (P < 0.001) used for admixture mapping. Sequence positions are aligned to the GRCh37/hg19 genome reference and are represented by the X-axis. [file 13195_2021_866_MOESM7_ESM.png]
