# Supplementary material for: Admixture mapping reveals the association between Native American ancestry at 3q13.11 and reduced risk of Alzheimer’s disease in Caribbean Hispanics
Source: Alzheimers Res Ther. 2021 Jul 3;13:122. doi: 10.1186/s13195-021-00866-9 (PMC8254995; doi:10.1186/s13195-021-00866-9)
Supplement: Supplementary file 2 — Additional file 2: File format: Microsoft Word .docx file. Title: Linkage disequilibrium blocks within regions with either genome-wide significant or suggestive evidence for association between local ancestry and Alzheimer’s disease. Description: Regions reaching at least suggestive evidence of association with Alzheimer’s disease are defined and the evidence for that association is summarized. Abbreviations: Chr = chromosome, Position = physical positions based on GRCh37/hg19 map, SNP = single nucleotide polymorphism, Effect size (95%CI): odds ratio for AD followed by the 95% confidence interval. [file 13195_2021_866_MOESM2_ESM.docx]

**Additional File 2. Linkage disequilibrium blocks within regions with either genome-wide significant or suggestive evidence for association between local ancestry and Alzheimer’s disease.** Abbreviations: Chr = chromosome, Position = physical positions based on GRCh37/hg19 map, SNP = single nucleotide polymorphism, Effect size (95%CI): odds ratio for AD followed by the 95% confidence interval.

| **Chr** | **#SNPs** | **Position** | **Lead SNP** | **Ancestry** | **OR (95%CI)** | ***P*** |
| --- | --- | --- | --- | --- | --- | --- |
| 2q22 .2 | 10 | 142,486,253 – 142,517,668 | rs13024316 | European | 1.28 (1.13;1.45) | 6.82E-04 |
| 2q22 .3 | 13 | 143,239,532 – 143,387,612 | rs7558786 | European | 1.27 (1.12;1.44) | 8.28E-04 |
| 3q13.11 | 33 | 103,747,624 – 104,082,176 | rs9858370 | Native American | 0.64 (0.51;0.80) | 1.28E-05 |
| 3q13.11 | 38 | 104,101,105 – 104,521,570 | rs9828652 | Native American | 0.64 (0.51;0.80) | 2.45E-05 |
| 3q13.11 | 21 | 104,530,166 – 104,642,165 | rs3996202 | Native American | 0.64 (0.51;0.80) | 2.21E-05 |
| 3q13.11 | 33 | 104,655,582 – 104,878,270 | rs6808509 | Native American | 0.63 (0.51;0.79) | 1.72E-05 |
| 3q13.11 | 10 | 104,881,936 – 104,998,768 | rs6770943 | Native American | 0.63 (0.50;0.78) | 7.94E-06 |
| 3q13.11 | 27 | 105,021,645 – 105,316,769 | rs6437577 | Native American | 0.62 (0.49;0.77) | 4.21E-06 |
| 3q13.11 | 5 | 105,328,255 – 105,370,343 | rs9822180 | Native American | 0.64 (0.51;0.80) | 1.27E-05 |
| 3q13.11 | 19 | 105,372,196 – 105,670,160 | rs11706098 | Native American | 0.65 (0.52;0.81) | 4.44E-05 |
| 3q13.11 | 6 | 105,673,976 – 105,694,636 | rs12629811 | Native American | 0.65 (0.52;0.81) | 4.29E-05 |
| 3q13.11 | 46 | 105,698,868 – 106,132,923 | rs9863266 | Native American | 0.60 (0.48;0.75) | 2.25E-06 |
| **3q13.11** | **25** | **106,139,801 – 106,283,820** | **rs10933849** | **Native American** | **0.58 (0.47;0.73)** | **8.76E-07** |
| 3q13.11 | 7 | 106,337,570 – 106,405,877 | rs2399106 | Native American | 0.62 (0.49;0.78) | 1.19E-05 |
| 3q13.11 | 38 | 106,985,680 – 107,297,709 | rs12489299 | Native American | 0.66 (0.53;0.83) | 2.26E-05 |
| 3q13.11 | 38 | 107,316,938 – 107,645,774 | rs9288851 | Native American | 0.66 (0.53;0.83) | 1.27E-05 |
| 3q13.11 | 19 | 107,646,728 – 107,725,831 | rs4855779 | Native American | 0.66 (0.53;0.83) | 2.89E-05 |
| 6q22. 31 | 34 | 123,548,997 – 123,838,033 | rs6940177 | Native American | 1.44 (1.19;1.75) | 9.54E-04 |
| 8q24.22 | 16 | 135,308,849 – 135,389,808 | rs4308771 | Native American | 1.36 (1.12;1.65) | 5.41E-04 |
| 8q24.22 | 42 | 135,409,616 – 135,575,624 | rs10808622 | Native American | 1.37 (1.13;1.66) | 7.87E-04 |
| 8q24.22 | 62 | 135,586,279 – 135,856,404 | rs7003440 | Native American | 1.37 (1.13;1.66) | 8.93E-04 |
| 9p21.3 | 30 | 22,207,037 – 22,544,804 | rs1679014 | Native American | 0.71 (0.57;0.88) | 8.29E-04 |
| 9p21.3 | 36 | 22,547,508 – 22,870,294 | rs4977586 | Native American | 0.70 (0.56;0.87) | 4.56E-04 |
| 14q12 | 19 | 32,485,703 – 32,701,864 | rs1952961 | African | 0.81 (0.71;0.93) | 7.24E-04 |
| 14q12 | 17 | 32,713,292 – 32,830,307 | rs7144586 | African | 0.82 (0.71;0.93) | 7.79E-04 |
| 14q12 | 19 | 32,842,066 - 32,908,280 | rs7494316 | African | 0.80 (0.70;0.92) | 9.07E-04 |
| 14q12 | 7 | 32,913,332 – 32,968,346 | rs7140949 | African | 0.81 (0.70;0.92) | 7.81E-04 |
| 14q12 | 10 | 32,981,484 – 33,033,695 | rs2145587 | African | 0.81 (0.70;0.92) | 8.83E-04 |
| 19p13.3 | 16 | 266,034 – 367,313 | rs2312724 | African | 1.28 (1.13;1.46) | 6.36E-04 |
| 19p13.3 | 5 | 372,661 – 389,873 | rs2303810 | African | 1.28 (1.12;1.46) | 7.50E-04 |
| 19p13.3 | 5 | 401,714 – 419,407 | rs7508251 | African | 1.28 (1.12;1.46) | 7.82E-04 |
| 19p13.3 | 5 | 420,755 – 499,978 | rs8112380 | African | 1.28 (1.12;1.45) | 8.34E-04 |
| 19p13.3 | 3 | 508,626 – 539,266 | rs4919908 | African | 1.27 (1.12;1.45) | 8.70E-04 |
| 19p13.3 | 3 | 552,650 – 554,919 | rs7247601 | African | 1.28 (1.13;1.46) | 6.42E-04 |
| 19p13.3 | 7 | 725,939 – 756,985 | rs3787017 | African | 1.29 (1.14;1.47) | 4.26E-04 |
| 19p13.3 | 7 | 789,890 – 844,020 | rs351967 | African | 1.28 (1.13;1.46) | 8.26E-04 |
| 19p13.3 | 3 | 1,475,391 – 1,505,874 | rs166451 | African | 1.29 (1.13;1.47) | 7.97E-04 |
