# Supplementary material for: Admixture mapping reveals the association between Native American ancestry at 3q13.11 and reduced risk of Alzheimer’s disease in Caribbean Hispanics
Source: Alzheimers Res Ther. 2021 Jul 3;13:122. doi: 10.1186/s13195-021-00866-9 (PMC8254995; doi:10.1186/s13195-021-00866-9)
Supplement: Supplementary file 8 — Additional file 8: File format: Microsoft Word .docx file. Title: Significant cis expression quantitative loci (eQTLs) for candidate genes within 3q13.11 region of interest. Description: Evidence that genotypes at a marker are associated with gene expression values. Results are filtered to only include those with false discovery rate < 0.05. Data are stored in the Synapse repository, Synapse ID: syn17015233, https://www.synapse.org/#!Synapse:syn17015233. Legend: Chr = chromosome, Position = GRCh37 position of the variant on chromosome 3, Z = Z statistic, FDR = false discovery rate, β = estimated effect size, A1 = allele 1, A2 = allele 2, A2freq = observed frequency of the A2 allele, Aup = allele associated with increased expression of the gene. [file 13195_2021_866_MOESM8_ESM.docx]

**Additional File 8: Significant *cis* expression quantitative loci (eQTLs) for candidate genes within 3q13.11 region of interest.** Results are filtered to only include those with false discovery rate < 0.05. Data are stored in the Synapse repository, Synapse ID: syn17015233, https://www.synapse.org/#!Synapse:syn17015233. Legend: Chr = chromosome, Position = GRCh37 position of the variant on chromosome 3, Z = Z statistic, FDR = false discovery rate, β = estimated effect size, A1 = allele 1, A2 = allele 2, A2freq = observed frequency of the A2 allele, Aup = allele associated with increased expression of the gene.

| **Position** | **SNP** | **Gene** | **Z** | **FDR** | **β** | **A1** | **A2** | **A2freq** | **Aup** |
| --- | --- | --- | --- | --- | --- | --- | --- | --- | --- |
| 104,097,336 | rs71325573 | *ALCAM* | -3.19 | 3.68E-02 | -0.2450 | A | G | 0.2042 | A |
| 104,101,105 | rs9828652 | *ALCAM* | -3.46 | 1.67E-02 | -0.2326 | G | T | 0.2943 | G |
| 104,102,258 | rs10933778 | *ALCAM* | -3.19 | 3.76E-02 | -0.2441 | C | T | 0.2033 | C |
| 104,103,488 | rs17369659 | *ALCAM* | -3.19 | 3.71E-02 | -0.2444 | A | G | 0.2031 | A |
| 104,106,616 | rs9876938 | *ALCAM* | -3.46 | 1.69E-02 | -0.2318 | G | T | 0.2932 | G |
| 104,106,617 | rs9838762 | *ALCAM* | -3.46 | 1.69E-02 | -0.2318 | A | G | 0.2932 | A |
| 104,107,435 | rs11720877 | *ALCAM* | 3.12 | 4.49E-02 | 0.1900 | C | T | 0.5043 | T |
| 104,231,516 | rs10049449 | *ALCAM* | 3.11 | 4.67E-02 | 0.1864 | G | A | 0.3907 | A |
| 104,234,104 | rs3892693 | *ALCAM* | 3.10 | 4.70E-02 | 0.1863 | C | T | 0.3915 | T |
| 104,234,146 | rs1107313 | *ALCAM* | 3.10 | 4.71E-02 | 0.1863 | A | C | 0.3916 | C |
| 104,234,207 | rs6437518 | *ALCAM* | 3.10 | 4.71E-02 | 0.1862 | G | A | 0.3915 | A |
| 104,234,545 | rs1913711 | *ALCAM* | 3.13 | 4.43E-02 | 0.1902 | A | C | 0.3860 | C |
| 104,235,019 | rs7429504 | *ALCAM* | 3.10 | 4.72E-02 | 0.1862 | A | G | 0.3916 | G |
| 104,235,352 | rs1588862 | *ALCAM* | 3.10 | 4.74E-02 | 0.1863 | G | A | 0.3909 | A |
| 104,235,365 | rs1588863 | *ALCAM* | 3.10 | 4.68E-02 | 0.1866 | G | A | 0.3908 | A |
| 104,235,414 | rs1588864 | *ALCAM* | 3.10 | 4.72E-02 | 0.1862 | T | G | 0.3910 | G |
| 104,235,681 | rs4364197 | *ALCAM* | 3.10 | 4.73E-02 | 0.1862 | C | T | 0.3908 | T |
| 104,235,682 | rs4257575 | *ALCAM* | 3.10 | 4.73E-02 | 0.1864 | A | G | 0.3908 | G |
| 104,238,223 | rs62260579 | *ALCAM* | 3.08 | 4.97E-02 | 0.1848 | C | T | 0.3903 | T |
| 104,288,181 | rs2961226 | *ALCAM* | 3.18 | 3.79E-02 | 0.1945 | A | G | 0.3591 | G |
| 104,307,403 | rs57284635 | *ALCAM* | -3.29 | 2.81E-02 | -0.2408 | C | A | 0.2150 | C |
| 104,308,811 | rs7614159 | *ALCAM* | -3.40 | 2.06E-02 | -0.2473 | G | A | 0.2220 | G |
| 104,310,358 | rs59312562 | *ALCAM* | -3.21 | 3.57E-02 | -0.2341 | G | A | 0.2139 | G |
| 104,313,914 | rs72984850 | *ALCAM* | -3.21 | 3.56E-02 | -0.2340 | G | A | 0.2142 | G |
| 104,326,292 | rs9809897 | *ALCAM* | -3.08 | 5.00E-02 | -0.2226 | A | C | 0.2202 | A |
| 104,329,096 | rs6802126 | *ALCAM* | -3.08 | 5.00E-02 | -0.2226 | G | A | 0.2202 | G |
| 104,334,753 | rs17189622 | *ALCAM* | -3.19 | 3.71E-02 | -0.2304 | A | C | 0.2234 | A |
| 104,348,000 | rs72986711 | *ALCAM* | -3.25 | 3.18E-02 | -0.2317 | G | A | 0.2338 | G |
| 104,348,709 | rs16850153 | *ALCAM* | -3.34 | 2.43E-02 | -0.2368 | G | T | 0.2371 | G |
| 104,352,305 | rs4145120 | *ALCAM* | -3.24 | 3.23E-02 | -0.2314 | C | A | 0.2335 | C |
| 104,354,201 | rs777941 | *ALCAM* | -3.37 | 2.21E-02 | -0.2290 | A | G | 0.2635 | A |
| 104,427,538 | rs2685043 | *ALCAM* | -3.13 | 4.36E-02 | -0.2514 | A | T | 0.1683 | A |
| 104,430,206 | rs2925862 | *ALCAM* | -3.12 | 4.44E-02 | -0.2510 | T | G | 0.1683 | T |
| 104,434,250 | rs777887 | *ALCAM* | -3.11 | 4.62E-02 | -0.2498 | T | C | 0.1683 | T |
| 104,441,440 | rs777893 | *ALCAM* | -3.11 | 4.60E-02 | -0.2498 | T | C | 0.1685 | T |
| 104,461,019 | rs777959 | *ALCAM* | -3.14 | 4.28E-02 | -0.2508 | A | G | 0.1716 | A |
| 104,907,697 | rs7626224 | *ALCAM* | 3.21 | 3.53E-02 | 0.2190 | G | T | 0.7459 | T |
| 105,002,129 | rs62262074 | *ALCAM* | -3.73 | 7.34E-03 | -0.2913 | C | G | 0.1755 | C |
| 105,002,602 | rs1259451 | *ALCAM* | -3.72 | 7.59E-03 | -0.2897 | C | A | 0.1755 | C |
| 105,003,196 | rs2692627 | *ALCAM* | -3.70 | 7.91E-03 | -0.2879 | G | T | 0.1755 | G |
| 105,004,853 | rs1795306 | *ALCAM* | -3.69 | 8.30E-03 | -0.2860 | T | C | 0.1755 | T |
| 105,007,051 | rs11709446 | *ALCAM* | 3.71 | 7.73E-03 | 0.2879 | C | T | 0.8245 | T |
| 105,009,538 | rs7631111 | *ALCAM* | 3.72 | 7.39E-03 | 0.2891 | C | T | 0.8245 | T |
| 105,010,535 | rs7634283 | *ALCAM* | 3.73 | 7.27E-03 | 0.2895 | C | T | 0.8245 | T |
| 105,012,459 | rs9816378 | *ALCAM* | 3.72 | 7.38E-03 | 0.2894 | A | G | 0.8246 | G |
| 105,013,778 | rs9879982 | *ALCAM* | 3.65 | 9.46E-03 | 0.2825 | T | C | 0.8205 | C |
| 105,014,277 | rs6437575 | *ALCAM* | 3.65 | 9.28E-03 | 0.2829 | C | T | 0.8205 | T |
| 105,014,318 | rs6437576 | *ALCAM* | 3.75 | 6.78E-03 | 0.2912 | G | A | 0.8246 | A |
| 105,015,073 | rs9878873 | *ALCAM* | 3.71 | 7.69E-03 | 0.2882 | A | G | 0.8241 | G |
| 105,021,383 | rs1920200 | *ALCAM* | 4.12 | 1.89E-03 | 0.3208 | C | T | 0.8303 | T |
| 105,021,940 | rs6437578 | *ALCAM* | 4.12 | 1.87E-03 | 0.3210 | A | G | 0.8303 | G |
| 105,023,561 | rs9809537 | *ALCAM* | 4.12 | 1.88E-03 | 0.3210 | C | G | 0.8304 | G |
| 105,024,155 | rs4361309 | *ALCAM* | 4.12 | 1.87E-03 | 0.3210 | C | T | 0.8304 | T |
| 105,025,156 | rs1920198 | *ALCAM* | 4.11 | 1.92E-03 | 0.3204 | T | C | 0.8303 | C |
| 105,026,035 | rs7628537 | *ALCAM* | 4.11 | 1.97E-03 | 0.3199 | A | G | 0.8303 | G |
| 105,027,045 | rs7621346 | *ALCAM* | 4.09 | 2.07E-03 | 0.3189 | T | C | 0.8301 | C |
| 105,027,500 | rs6786845 | *ALCAM* | 4.09 | 2.10E-03 | 0.3187 | A | G | 0.8303 | G |
| 105,030,685 | rs6786056 | *ALCAM* | 4.05 | 2.42E-03 | 0.3156 | T | C | 0.8301 | C |
| 105,072,875 | rs62262152 | *ALCAM* | 3.09 | 4.86E-02 | 0.1974 | G | C | 0.6947 | C |
| 105,072,879 | rs62262153 | *ALCAM* | 3.12 | 4.49E-02 | 0.2000 | T | C | 0.6915 | C |
| 105,073,627 | rs17795071 | *ALCAM* | -3.55 | 1.31E-02 | -0.2356 | C | G | 0.2469 | C |
| 105,074,864 | rs56161833 | *ALCAM* | -3.62 | 1.03E-02 | -0.2400 | G | A | 0.2479 | G |
| 105,082,175 | rs73177458 | *ALCAM* | -4.24 | 1.21E-03 | -0.3839 | G | A | 0.1178 | G |
| 105,086,826 | rs73177459 | *ALCAM* | -4.18 | 1.51E-03 | -0.3324 | A | G | 0.1574 | A |
| 105,088,435 | rs34029528 | *ALCAM* | -4.30 | 9.47E-04 | -0.3504 | T | G | 0.1530 | T |
| 105,094,590 | rs73177465 | *ALCAM* | -4.26 | 1.11E-03 | -0.3521 | C | T | 0.1440 | C |
| 105,095,612 | rs6771935 | *ALCAM* | -4.20 | 1.40E-03 | -0.3349 | G | A | 0.1570 | G |
| 105,099,000 | rs73177470 | *ALCAM* | -4.22 | 1.31E-03 | -0.3362 | T | C | 0.1573 | T |
| 105,103,199 | rs11716035 | *ALCAM* | -4.33 | 8.56E-04 | -0.3515 | G | A | 0.1521 | G |
| 105,105,183 | rs73177478 | *ALCAM* | -4.33 | 8.37E-04 | -0.3518 | G | C | 0.1523 | G |
| 105,108,867 | rs6797043 | *ALCAM* | -4.89 | 8.24E-05 | -0.3429 | T | C | 0.2309 | T |
| 105,111,373 | rs75186868 | *ALCAM* | -4.31 | 9.30E-04 | -0.3553 | C | T | 0.1442 | C |
| 105,114,923 | rs73177488 | *ALCAM* | -4.89 | 8.46E-05 | -0.3426 | A | C | 0.2312 | A |
| 105,115,370 | rs73177489 | *ALCAM* | -4.31 | 9.08E-04 | -0.3557 | G | A | 0.1441 | G |
| 105,118,968 | rs73177494 | *ALCAM* | -4.38 | 7.10E-04 | -0.3483 | G | A | 0.1558 | G |
| 105,120,836 | rs66965318 | *ALCAM* | -4.32 | 8.90E-04 | -0.3560 | A | T | 0.1441 | A |
| 105,121,987 | rs11719817 | *ALCAM* | -4.32 | 8.87E-04 | -0.3560 | C | T | 0.1440 | C |
| 105,133,137 | rs73179378 | *ALCAM* | -4.31 | 9.33E-04 | -0.3548 | A | G | 0.1440 | A |
| 105,134,768 | rs73179384 | *ALCAM* | -4.48 | 4.64E-04 | -0.3636 | T | C | 0.1511 | T |
| 105,136,005 | rs2895300 | *ALCAM* | -4.31 | 9.15E-04 | -0.3553 | C | A | 0.1440 | C |
| 105,138,520 | rs74709034 | *ALCAM* | -4.30 | 9.44E-04 | -0.3546 | G | A | 0.1435 | G |
| 105,145,335 | rs6786815 | *ALCAM* | -4.37 | 7.19E-04 | -0.3479 | C | A | 0.1550 | C |
| 105,147,072 | rs11714277 | *ALCAM* | -4.37 | 7.14E-04 | -0.3480 | G | T | 0.1550 | G |
| 105,147,202 | rs11718405 | *ALCAM* | -4.37 | 7.14E-04 | -0.3480 | T | C | 0.1550 | T |
| 105,147,664 | rs73181374 | *ALCAM* | -4.31 | 9.06E-04 | -0.3554 | T | A | 0.1434 | T |
| 105,148,628 | rs73181376 | *ALCAM* | -4.38 | 7.10E-04 | -0.3480 | C | T | 0.1550 | C |
| 105,149,832 | rs73181380 | *ALCAM* | -4.32 | 9.00E-04 | -0.3555 | A | G | 0.1434 | A |
| 105,150,803 | rs73181387 | *ALCAM* | -4.49 | 4.48E-04 | -0.3641 | A | C | 0.1505 | A |
| 105,155,209 | rs112158087 | *ALCAM* | -4.38 | 6.90E-04 | -0.3483 | C | T | 0.1550 | C |
| 105,156,468 | rs11718457 | *ALCAM* | -4.32 | 8.79E-04 | -0.3557 | C | G | 0.1433 | C |
| 105,159,106 | rs76110958 | *ALCAM* | -4.39 | 6.79E-04 | -0.3484 | G | A | 0.1550 | G |
| 105,159,810 | rs73181395 | *ALCAM* | -4.39 | 6.78E-04 | -0.3485 | G | A | 0.1550 | G |
| 105,160,000 | rs73181396 | *ALCAM* | -4.39 | 6.78E-04 | -0.3485 | G | A | 0.1550 | G |
| 105,160,397 | rs73181397 | *ALCAM* | -4.32 | 8.76E-04 | -0.3556 | A | T | 0.1434 | A |
| 105,160,788 | rs6771987 | *ALCAM* | -4.39 | 6.75E-04 | -0.3485 | C | T | 0.1550 | C |
| 105,161,003 | rs73181401 | *ALCAM* | -4.39 | 6.75E-04 | -0.3485 | G | A | 0.1550 | G |
| 105,163,244 | rs73183103 | *ALCAM* | -4.39 | 6.68E-04 | -0.3486 | G | C | 0.1550 | G |
| 105,164,428 | rs73183108 | *ALCAM* | -4.39 | 6.64E-04 | -0.3487 | A | G | 0.1550 | A |
| 105,164,872 | rs1955135 | *ALCAM* | -4.39 | 6.64E-04 | -0.3487 | A | C | 0.1550 | A |
| 105,165,692 | rs73183112 | *ALCAM* | -4.39 | 6.61E-04 | -0.3487 | T | C | 0.1550 | T |
| 105,166,451 | rs73183114 | *ALCAM* | -4.41 | 6.20E-04 | -0.3518 | G | A | 0.1534 | G |
| 105,166,455 | rs73183115 | *ALCAM* | -4.39 | 6.70E-04 | -0.3486 | A | G | 0.1548 | A |
| 105,168,098 | rs11710166 | *ALCAM* | -4.40 | 6.57E-04 | -0.3488 | C | A | 0.1550 | C |
| 105,168,203 | rs11714275 | *ALCAM* | -4.50 | 4.23E-04 | -0.3645 | T | C | 0.1504 | T |
| 105,169,639 | rs73183185 | *ALCAM* | -4.38 | 7.02E-04 | -0.3476 | G | T | 0.1550 | G |
| 105,169,703 | rs73183186 | *ALCAM* | -4.32 | 8.80E-04 | -0.3552 | G | A | 0.1434 | G |
| 105,169,774 | rs73183187 | *ALCAM* | -4.38 | 7.08E-04 | -0.3475 | A | G | 0.1550 | A |
| 105,169,822 | rs73183188 | *ALCAM* | -4.38 | 7.10E-04 | -0.3474 | G | A | 0.1550 | G |
| 105,170,646 | rs1898655 | *ALCAM* | -4.37 | 7.21E-04 | -0.3472 | C | A | 0.1550 | C |
| 105,172,069 | rs113549473 | *ALCAM* | -4.36 | 7.57E-04 | -0.3463 | A | C | 0.1551 | A |
| 105,172,851 | rs59322590 | *ALCAM* | -4.35 | 7.85E-04 | -0.3456 | G | A | 0.1551 | G |
| 105,172,883 | rs61522255 | *ALCAM* | -4.35 | 7.87E-04 | -0.3456 | A | C | 0.1551 | A |
| 105,173,574 | rs113360413 | *ALCAM* | -3.43 | 1.85E-02 | -0.2666 | G | A | 0.1737 | G |
| 105,173,638 | rs111669332 | *ALCAM* | -4.34 | 8.10E-04 | -0.3450 | A | G | 0.1551 | A |
| 105,174,202 | rs138904061 | *ALCAM* | -4.17 | 1.54E-03 | -0.3298 | T | C | 0.1615 | T |
| 105,174,678 | rs73183198 | *ALCAM* | -4.16 | 1.62E-03 | -0.3286 | A | T | 0.1615 | A |
| 105,176,408 | rs73185106 | *ALCAM* | -4.09 | 2.10E-03 | -0.3228 | A | T | 0.1619 | A |
| 105,177,679 | rs6794273 | *ALCAM* | -4.14 | 1.76E-03 | -0.3266 | G | A | 0.1616 | G |
| 105,178,396 | rs73185114 | *ALCAM* | -4.14 | 1.76E-03 | -0.3266 | C | T | 0.1616 | C |
| 105,179,024 | rs73185115 | *ALCAM* | -4.14 | 1.76E-03 | -0.3266 | C | T | 0.1616 | C |
| 105,181,233 | rs11710857 | *ALCAM* | -4.24 | 1.22E-03 | -0.3408 | A | T | 0.1570 | A |
| 105,184,060 | rs75124880 | *ALCAM* | -4.24 | 1.22E-03 | -0.3408 | G | T | 0.1570 | G |
| 105,185,279 | rs57986655 | *ALCAM* | -4.24 | 1.21E-03 | -0.3409 | G | A | 0.1570 | G |
| 105,192,308 | rs11713535 | *ALCAM* | -4.29 | 1.01E-03 | -0.3528 | T | C | 0.1435 | T |
| 105,195,524 | rs73189021 | *ALCAM* | -4.23 | 1.24E-03 | -0.3405 | G | A | 0.1571 | G |
| 105,200,951 | rs73189028 | *ALCAM* | -4.27 | 1.09E-03 | -0.3465 | G | A | 0.1555 | G |
| 105,204,769 | rs73189034 | *ALCAM* | -4.27 | 1.09E-03 | -0.3466 | A | G | 0.1555 | A |
| 105,205,066 | rs6769329 | *ALCAM* | -3.58 | 1.19E-02 | -0.2147 | C | A | 0.4684 | C |
| 105,208,662 | rs6804472 | *ALCAM* | -3.58 | 1.17E-02 | -0.2150 | A | G | 0.4684 | A |
| 105,209,768 | rs11715283 | *ALCAM* | -4.33 | 8.43E-04 | -0.3603 | T | C | 0.1412 | T |
| 105,210,206 | rs12490553 | *ALCAM* | -3.58 | 1.17E-02 | -0.2150 | C | T | 0.4684 | C |
| 105,213,861 | rs73189046 | *ALCAM* | -4.43 | 5.79E-04 | -0.3987 | G | A | 0.1184 | G |
| 105,217,466 | rs73189060 | *ALCAM* | -4.29 | 1.01E-03 | -0.3483 | G | A | 0.1554 | G |
| 105,222,024 | rs76416303 | *ALCAM* | -4.29 | 9.85E-04 | -0.3486 | C | G | 0.1556 | C |
| 105,224,595 | rs74572454 | *ALCAM* | -4.30 | 9.55E-04 | -0.3491 | A | G | 0.1563 | A |
| 105,225,340 | rs2200473 | *ALCAM* | -4.30 | 9.51E-04 | -0.3492 | A | G | 0.1563 | A |
| 105,229,549 | rs978436 | *ALCAM* | -4.31 | 9.29E-04 | -0.3494 | G | A | 0.1563 | G |
| 105,233,814 | rs73192717 | *ALCAM* | -4.31 | 9.15E-04 | -0.3494 | A | C | 0.1564 | A |
| 105,235,627 | rs73192730 | *ALCAM* | -4.37 | 7.15E-04 | -0.3627 | A | G | 0.1415 | A |
| 105,236,993 | rs73192733 | *ALCAM* | -4.37 | 7.19E-04 | -0.3626 | C | T | 0.1416 | C |
| 105,237,998 | rs73192737 | *ALCAM* | -4.35 | 7.80E-04 | -0.3609 | A | G | 0.1427 | A |
| 105,238,075 | rs78947472 | *ALCAM* | -4.35 | 7.81E-04 | -0.3608 | C | T | 0.1429 | C |
| 105,244,271 | rs73192751 | *ALCAM* | -4.44 | 5.51E-04 | -0.3691 | A | G | 0.1425 | A |
| 105,244,693 | rs73192754 | *ALCAM* | -4.44 | 5.49E-04 | -0.3692 | G | A | 0.1425 | G |
| 105,247,736 | rs6793123 | *ALCAM* | 3.33 | 2.54E-02 | 0.2048 | C | A | 0.6689 | A |
| 105,248,087 | rs12630003 | *ALCAM* | 3.51 | 1.44E-02 | 0.2266 | A | G | 0.3383 | G |
| 105,249,211 | rs3772556 | *ALCAM* | 3.55 | 1.27E-02 | 0.2325 | C | T | 0.7163 | T |
| 105,251,445 | rs73192770 | *ALCAM* | -4.42 | 5.85E-04 | -0.3683 | C | G | 0.1417 | C |
| 105,251,644 | rs67408187 | *ALCAM* | 3.53 | 1.36E-02 | 0.2276 | G | C | 0.3385 | C |
| 105,251,782 | rs66875175 | *ALCAM* | 3.53 | 1.36E-02 | 0.2276 | A | G | 0.3385 | G |
| 105,251,962 | rs55925195 | *ALCAM* | 3.53 | 1.36E-02 | 0.2275 | A | G | 0.3385 | G |
| 105,252,687 | rs2399049 | *ALCAM* | 4.36 | 7.48E-04 | 0.3528 | G | A | 0.8480 | A |
| 105,252,950 | rs73175407 | *ALCAM* | -4.42 | 5.89E-04 | -0.3681 | T | C | 0.1416 | T |
| 105,258,334 | rs517415 | *ALCAM* | 4.37 | 7.29E-04 | 0.3530 | G | A | 0.8483 | A |
| 105,260,798 | rs598425 | *ALCAM* | 4.37 | 7.32E-04 | 0.3530 | G | T | 0.8483 | T |
| 105,262,148 | rs526297 | *ALCAM* | 4.29 | 9.94E-04 | 0.3566 | C | T | 0.8572 | T |
| 105,263,108 | rs481177 | *ALCAM* | 4.37 | 7.35E-04 | 0.3529 | G | A | 0.8484 | A |
| 105,263,258 | rs73175433 | *ALCAM* | -4.42 | 6.00E-04 | -0.3676 | T | C | 0.1414 | T |
| 105,264,482 | rs73175435 | *ALCAM* | -4.42 | 5.98E-04 | -0.3677 | G | A | 0.1414 | G |
| 105,266,484 | rs73175442 | *ALCAM* | -4.42 | 6.06E-04 | -0.3675 | T | G | 0.1409 | T |
| 105,267,436 | rs3772547 | *ALCAM* | -4.42 | 6.05E-04 | -0.3674 | A | G | 0.1409 | A |
| 105,267,500 | rs495691 | *ALCAM* | 4.31 | 9.21E-04 | 0.3270 | G | T | 0.8063 | T |
| 105,267,541 | rs587467 | *ALCAM* | 3.66 | 9.25E-03 | 0.2305 | C | A | 0.6750 | A |
| 105,267,827 | rs3772544 | *ALCAM* | -4.41 | 6.08E-04 | -0.3674 | G | C | 0.1409 | G |
| 105,270,017 | rs73175449 | *ALCAM* | -4.41 | 6.11E-04 | -0.3674 | G | A | 0.1409 | G |
| 105,270,477 | rs73175451 | *ALCAM* | -4.41 | 6.11E-04 | -0.3674 | C | T | 0.1409 | C |
| 105,274,780 | rs3772542 | *ALCAM* | -4.41 | 6.15E-04 | -0.3675 | G | A | 0.1409 | G |
| 105,276,969 | rs474355 | *ALCAM* | 4.26 | 1.13E-03 | 0.3340 | A | G | 0.8156 | G |
| 105,278,861 | rs73175463 | *ALCAM* | -4.41 | 6.25E-04 | -0.3673 | T | C | 0.1409 | T |
| 105,282,822 | rs146325884 | *ALCAM* | -4.41 | 6.27E-04 | -0.3675 | C | T | 0.1406 | C |
| 105,296,313 | rs567193 | *ALCAM* | 4.26 | 1.13E-03 | 0.3340 | G | C | 0.8168 | C |
| 105,297,267 | rs73175491 | *ALCAM* | -4.40 | 6.48E-04 | -0.3676 | T | C | 0.1396 | T |
| 105,297,450 | rs666474 | *ALCAM* | 4.25 | 1.16E-03 | 0.3337 | G | A | 0.8169 | A |
| 105,311,399 | rs141451299 | *ALCAM* | -4.52 | 3.97E-04 | -0.4104 | C | T | 0.1222 | C |
| 105,313,124 | rs73177609 | *ALCAM* | -4.48 | 4.70E-04 | -0.3918 | C | A | 0.1319 | C |
| 105,314,429 | rs73177619 | *ALCAM* | -4.46 | 5.05E-04 | -0.3913 | G | A | 0.1317 | G |
| 105,324,209 | rs60180236 | *ALCAM* | -3.81 | 5.55E-03 | -0.3317 | T | C | 0.1479 | T |
| 105,324,871 | rs2120582 | *ALCAM* | -3.90 | 4.05E-03 | -0.3399 | T | C | 0.1474 | T |
| 105,326,164 | rs73179804 | *ALCAM* | -3.77 | 6.32E-03 | -0.3289 | C | T | 0.1476 | C |
| 105,327,909 | rs78945584 | *ALCAM* | -3.83 | 5.18E-03 | -0.3348 | T | C | 0.1458 | T |
| 105,330,656 | rs1522291 | *ALCAM* | -3.83 | 5.22E-03 | -0.3198 | A | C | 0.1614 | A |
| 105,332,349 | rs73179817 | *ALCAM* | -3.83 | 5.25E-03 | -0.3229 | G | T | 0.1604 | G |
| 107,510,457 | rs6437745 | *BBX* | -3.25 | 3.14E-02 | -0.2603 | G | A | 0.8506 | G |
| 107,527,596 | rs9831296 | *BBX* | 3.35 | 2.39E-02 | 0.2650 | A | C | 0.1516 | C |
| 107,529,255 | rs10804464 | *BBX* | 3.35 | 2.40E-02 | 0.2649 | G | A | 0.1516 | A |
| 107,530,735 | rs9847515 | *BBX* | 3.14 | 4.31E-02 | 0.2495 | A | C | 0.1512 | C |
| 107,532,150 | rs17813292 | *BBX* | 3.13 | 4.34E-02 | 0.2493 | A | G | 0.1511 | G |
| 107,536,562 | rs9813554 | *BBX* | 3.12 | 4.44E-02 | 0.2485 | C | A | 0.1512 | A |
| 107,538,202 | rs9843504 | *BBX* | 3.12 | 4.48E-02 | 0.2482 | T | C | 0.1516 | C |
| 107,540,714 | rs6808795 | *BBX* | 3.16 | 4.03E-02 | 0.1989 | G | A | 0.3507 | A |
| 107,576,434 | rs9838263 | *BBX* | 3.64 | 9.61E-03 | 0.2577 | G | A | 0.2270 | A |
| 107,577,262 | rs9843526 | *BBX* | 3.66 | 8.97E-03 | 0.2592 | G | C | 0.2272 | C |
| 107,579,115 | rs17813802 | *BBX* | 3.69 | 8.39E-03 | 0.2607 | G | A | 0.2274 | A |
| 107,581,048 | rs9864366 | *BBX* | 3.66 | 9.14E-03 | 0.2589 | G | A | 0.2275 | A |
| 107,583,197 | rs9875001 | *BBX* | 3.72 | 7.43E-03 | 0.2620 | C | T | 0.2295 | T |
| 107,584,575 | rs9881309 | *BBX* | 3.56 | 1.25E-02 | 0.2536 | G | A | 0.2272 | A |
| 107,585,109 | rs13322960 | *BBX* | 3.54 | 1.31E-02 | 0.2471 | G | T | 0.2380 | T |
| 107,587,513 | rs28668039 | *BBX* | 3.59 | 1.13E-02 | 0.2502 | A | G | 0.2382 | G |
| 107,588,505 | rs9818603 | *BBX* | 3.55 | 1.27E-02 | 0.2478 | T | A | 0.2383 | A |
| 107,589,681 | rs9879639 | *BBX* | 3.60 | 1.12E-02 | 0.2507 | C | T | 0.2388 | T |
| 107,591,674 | rs11707020 | *BBX* | 3.60 | 1.09E-02 | 0.2511 | G | A | 0.2386 | A |
| 107,592,185 | rs11707950 | *BBX* | 3.60 | 1.09E-02 | 0.2511 | G | A | 0.2386 | A |
| 107,595,782 | rs62264009 | *BBX* | 3.51 | 1.46E-02 | 0.2481 | C | T | 0.2272 | T |
| 107,596,954 | rs6773129 | *BBX* | 3.46 | 1.71E-02 | 0.2437 | A | G | 0.2300 | G |
| 105,373,929 | rs17202230 | *CBLB* | -3.27 | 3.00E-02 | -0.2164 | T | C | 0.2722 | T |
| 105,374,814 | rs9657915 | *CBLB* | -3.21 | 3.49E-02 | -0.2110 | A | G | 0.2737 | A |
| 105,375,252 | rs9657913 | *CBLB* | -3.20 | 3.60E-02 | -0.2120 | A | T | 0.2719 | A |
| 105,377,515 | rs1042852 | *CBLB* | -3.27 | 3.00E-02 | -0.2143 | C | T | 0.2732 | C |
| 105,378,473 | rs28532512 | *CBLB* | -3.27 | 2.97E-02 | -0.2145 | G | A | 0.2732 | G |
| 105,379,946 | rs57735492 | *CBLB* | 3.90 | 4.05E-03 | 0.4373 | C | T | 0.0756 | T |
| 105,381,361 | rs13083716 | *CBLB* | -3.29 | 2.83E-02 | -0.2153 | G | A | 0.2736 | G |
| 105,381,695 | rs13084260 | *CBLB* | -3.29 | 2.82E-02 | -0.2154 | G | C | 0.2735 | G |
| 105,387,224 | rs34520191 | *CBLB* | -3.32 | 2.61E-02 | -0.2167 | T | G | 0.2735 | T |
| 105,387,230 | rs35834008 | *CBLB* | -3.32 | 2.61E-02 | -0.2167 | C | A | 0.2735 | C |
| 105,388,590 | rs9877805 | *CBLB* | -3.08 | 4.99E-02 | -0.2168 | C | T | 0.2236 | C |
| 105,388,605 | rs9878058 | *CBLB* | -3.19 | 3.70E-02 | -0.2259 | G | A | 0.2221 | G |
| 105,392,150 | rs9860539 | *CBLB* | -3.08 | 4.95E-02 | -0.2166 | A | C | 0.2235 | A |
| 105,392,392 | rs1530420 | *CBLB* | -3.34 | 2.44E-02 | -0.2176 | T | C | 0.2734 | T |
| 105,393,335 | rs13317635 | *CBLB* | -3.08 | 4.94E-02 | -0.2165 | G | C | 0.2235 | G |
| 105,398,093 | rs7624530 | *CBLB* | -3.21 | 3.55E-02 | -0.2261 | G | A | 0.2222 | G |
| 105,398,552 | rs35748894 | *CBLB* | -3.10 | 4.77E-02 | -0.2170 | G | A | 0.2237 | G |
| 105,401,406 | rs2301051 | *CBLB* | -3.32 | 2.58E-02 | -0.2172 | A | T | 0.2718 | A |
| 105,401,507 | rs2301050 | *CBLB* | -3.38 | 2.17E-02 | -0.2192 | C | T | 0.2735 | C |
| 105,401,989 | rs2301048 | *CBLB* | 3.96 | 3.30E-03 | 0.4394 | C | T | 0.0755 | T |
| 105,402,744 | rs2120585 | *CBLB* | -3.49 | 1.57E-02 | -0.2266 | A | G | 0.2721 | A |
| 105,403,010 | rs3772540 | *CBLB* | 3.97 | 3.26E-03 | 0.4395 | C | T | 0.0755 | T |
| 105,403,685 | rs9816486 | *CBLB* | -3.39 | 2.10E-02 | -0.2195 | A | G | 0.2736 | A |
| 105,405,280 | rs2284993 | *CBLB* | -3.40 | 2.07E-02 | -0.2197 | A | G | 0.2736 | A |
| 105,406,468 | rs13315174 | *CBLB* | -3.23 | 3.33E-02 | -0.2266 | G | A | 0.2224 | G |
| 105,407,296 | rs3772537 | *CBLB* | -3.23 | 3.32E-02 | -0.2267 | C | G | 0.2225 | C |
| 105,407,561 | rs9840891 | *CBLB* | -3.12 | 4.48E-02 | -0.2177 | T | C | 0.2240 | T |
| 105,410,694 | rs3821488 | *CBLB* | -3.13 | 4.43E-02 | -0.2179 | C | T | 0.2240 | C |
| 105,411,449 | rs2244737 | *CBLB* | -3.41 | 1.97E-02 | -0.2203 | A | G | 0.2737 | A |
| 105,417,893 | rs9870097 | *CBLB* | -3.52 | 1.43E-02 | -0.2279 | T | C | 0.2724 | T |
| 105,431,035 | rs2301036 | *CBLB* | 3.99 | 3.00E-03 | 0.4399 | T | C | 0.0755 | C |
| 105,437,781 | rs3772531 | *CBLB* | -3.09 | 4.83E-02 | -0.1974 | T | C | 0.2832 | T |
| 105,438,806 | rs7624400 | *CBLB* | -3.81 | 5.55E-03 | -0.3847 | G | A | 0.8994 | G |
| 105,441,133 | rs1075739 | *CBLB* | 3.81 | 5.65E-03 | 0.3844 | T | C | 0.1005 | C |
| 105,441,542 | rs3772527 | *CBLB* | -3.09 | 4.84E-02 | -0.1974 | T | C | 0.2833 | T |
| 105,441,707 | rs3772526 | *CBLB* | -3.09 | 4.83E-02 | -0.1974 | A | G | 0.2833 | A |
| 105,442,953 | rs894937 | *CBLB* | -3.19 | 3.74E-02 | -0.2043 | T | C | 0.2818 | T |
| 105,443,465 | rs12635232 | *CBLB* | 3.80 | 5.78E-03 | 0.3837 | G | C | 0.1005 | C |
| 105,447,746 | rs1867189 | *CBLB* | -3.19 | 3.75E-02 | -0.2042 | G | A | 0.2820 | G |
| 105,447,765 | rs16851523 | *CBLB* | 3.80 | 5.83E-03 | 0.3834 | T | C | 0.1004 | C |
| 105,449,793 | rs9876767 | *CBLB* | -3.09 | 4.88E-02 | -0.1973 | T | G | 0.2835 | T |
| 105,449,986 | rs9880861 | *CBLB* | -3.09 | 4.88E-02 | -0.1972 | T | C | 0.2835 | T |
| 105,450,301 | rs34896116 | *CBLB* | -3.19 | 3.77E-02 | -0.2042 | T | A | 0.2820 | T |
| 105,450,512 | rs12639271 | *CBLB* | 3.80 | 5.77E-03 | 0.3836 | T | C | 0.1004 | C |
| 105,454,880 | rs16851539 | *CBLB* | 3.80 | 5.82E-03 | 0.3834 | A | C | 0.1003 | C |
| 105,455,161 | rs16844736 | *CBLB* | 3.99 | 2.99E-03 | 0.4395 | T | C | 0.0753 | C |
| 105,456,983 | rs13065142 | *CBLB* | -3.16 | 4.02E-02 | -0.2026 | T | C | 0.2825 | T |
| 105,457,377 | rs9789988 | *CBLB* | 3.80 | 5.83E-03 | 0.3833 | T | C | 0.1003 | C |
| 105,460,388 | rs60295307 | *CBLB* | 3.82 | 5.31E-03 | 0.3862 | C | T | 0.1011 | T |
| 105,461,728 | rs74984353 | *CBLB* | 3.99 | 3.00E-03 | 0.4395 | C | T | 0.0752 | T |
| 105,461,914 | rs79879407 | *CBLB* | 3.99 | 3.00E-03 | 0.4395 | G | A | 0.0752 | A |
| 105,465,404 | rs2197208 | *CBLB* | 3.80 | 5.70E-03 | 0.3840 | C | T | 0.1002 | T |
| 105,467,668 | rs60619938 | *CBLB* | 3.81 | 5.58E-03 | 0.3847 | C | T | 0.1002 | T |
| 105,469,882 | rs16851563 | *CBLB* | 3.82 | 5.44E-03 | 0.3856 | T | C | 0.1003 | C |
| 105,470,134 | rs1947000 | *CBLB* | 3.82 | 5.46E-03 | 0.3856 | T | G | 0.1003 | G |
| 105,470,758 | rs2399055 | *CBLB* | -3.18 | 3.85E-02 | -0.2038 | T | C | 0.2826 | T |
| 105,472,390 | rs16851570 | *CBLB* | 3.82 | 5.39E-03 | 0.3861 | T | C | 0.1002 | C |
| 105,475,655 | rs12493181 | *CBLB* | -3.12 | 4.45E-02 | -0.1998 | T | C | 0.2835 | T |
| 105,475,939 | rs13093260 | *CBLB* | -3.23 | 3.38E-02 | -0.2071 | C | T | 0.2820 | C |
| 105,478,162 | rs2083758 | *CBLB* | -3.11 | 4.58E-02 | -0.1992 | A | G | 0.2839 | A |
| 105,480,602 | rs4321559 | *CBLB* | -3.24 | 3.21E-02 | -0.2083 | C | T | 0.2818 | C |
| 105,480,833 | rs11929289 | *CBLB* | -3.25 | 3.19E-02 | -0.2085 | T | C | 0.2818 | T |
| 105,482,422 | rs11915926 | *CBLB* | -3.13 | 4.40E-02 | -0.2002 | T | C | 0.2836 | T |
| 105,484,620 | rs6796643 | *CBLB* | -3.26 | 3.08E-02 | -0.2094 | C | T | 0.2817 | C |
| 105,485,555 | rs1838046 | *CBLB* | 3.37 | 2.22E-02 | 0.2044 | T | C | 0.6796 | C |
| 105,486,255 | rs3772515 | *CBLB* | -3.17 | 3.98E-02 | -0.2027 | T | C | 0.2832 | T |
| 105,487,849 | rs6773661 | *CBLB* | -3.15 | 4.20E-02 | -0.2013 | T | C | 0.2835 | T |
| 105,487,964 | rs140128461 | *CBLB* | 4.00 | 2.85E-03 | 0.4411 | C | T | 0.0752 | T |
| 105,488,134 | rs139969708 | *CBLB* | 4.01 | 2.85E-03 | 0.4411 | C | T | 0.0755 | T |
| 105,488,572 | rs3772513 | *CBLB* | -3.09 | 4.86E-02 | -0.1993 | A | G | 0.2817 | A |
| 105,492,787 | rs7649520 | *CBLB* | -3.10 | 4.72E-02 | -0.2002 | C | T | 0.2810 | C |
| 105,492,848 | rs74848961 | *CBLB* | 4.00 | 2.88E-03 | 0.4409 | C | T | 0.0753 | T |
| 105,493,286 | rs7619647 | *CBLB* | -3.25 | 3.12E-02 | -0.2092 | T | C | 0.2813 | T |
| 105,496,135 | rs75994561 | *CBLB* | 3.88 | 4.33E-03 | 0.4246 | C | A | 0.0764 | A |
| 105,498,117 | rs74735269 | *CBLB* | 3.88 | 4.35E-03 | 0.4244 | A | G | 0.0765 | G |
| 105,499,206 | rs78818427 | *CBLB* | 3.88 | 4.37E-03 | 0.4243 | A | G | 0.0765 | G |
| 105,504,129 | rs10460808 | *CBLB* | 3.88 | 4.38E-03 | 0.4242 | A | C | 0.0765 | C |
| 105,510,064 | rs80190767 | *CBLB* | 3.87 | 4.47E-03 | 0.4236 | C | T | 0.0766 | T |
| 105,510,314 | rs12637809 | *CBLB* | 3.72 | 7.56E-03 | 0.3704 | C | T | 0.1015 | T |
| 105,511,196 | rs1992521 | *CBLB* | -3.88 | 4.45E-03 | -0.4237 | T | C | 0.9232 | T |
| 105,514,529 | rs80010410 | *CBLB* | 3.86 | 4.69E-03 | 0.4222 | T | C | 0.0766 | C |
| 105,516,159 | rs79353459 | *CBLB* | 3.87 | 4.49E-03 | 0.4234 | A | G | 0.0765 | G |
| 105,516,352 | rs78055346 | *CBLB* | 3.87 | 4.49E-03 | 0.4234 | G | A | 0.0762 | A |
| 105,516,844 | rs76661759 | *CBLB* | 3.87 | 4.49E-03 | 0.4234 | G | A | 0.0767 | A |
| 105,517,328 | rs12633654 | *CBLB* | 3.71 | 7.62E-03 | 0.3701 | A | G | 0.1013 | G |
| 105,519,242 | rs59611997 | *CBLB* | 3.71 | 7.63E-03 | 0.3701 | G | A | 0.1015 | A |
| 105,520,466 | rs77351708 | *CBLB* | 3.87 | 4.48E-03 | 0.4235 | C | T | 0.0762 | T |
| 105,523,135 | rs111776667 | *CBLB* | 3.71 | 7.64E-03 | 0.3701 | C | T | 0.1014 | T |
| 105,523,216 | rs116338220 | *CBLB* | 3.71 | 7.62E-03 | 0.3701 | C | T | 0.1013 | T |
| 105,526,724 | rs1561922 | *CBLB* | 3.71 | 7.62E-03 | 0.3701 | T | C | 0.1015 | C |
| 105,527,222 | rs73854891 | *CBLB* | 3.71 | 7.62E-03 | 0.3701 | T | A | 0.1015 | A |
| 105,528,341 | rs79091188 | *CBLB* | 3.87 | 4.49E-03 | 0.4234 | C | T | 0.0765 | T |
| 105,533,713 | rs16851630 | *CBLB* | 3.71 | 7.64E-03 | 0.3702 | G | A | 0.1015 | A |
| 105,538,485 | rs73854894 | *CBLB* | 3.71 | 7.66E-03 | 0.3701 | A | G | 0.1016 | G |
| 105,539,089 | rs78137614 | *CBLB* | 3.87 | 4.50E-03 | 0.4235 | G | A | 0.0767 | A |
| 105,541,105 | rs59534462 | *CBLB* | 3.71 | 7.66E-03 | 0.3701 | G | C | 0.1016 | C |
| 105,543,370 | rs76618846 | *CBLB* | 3.87 | 4.48E-03 | 0.4236 | G | A | 0.0768 | A |
| 105,543,982 | rs73857222 | *CBLB* | 3.72 | 7.60E-03 | 0.3703 | T | C | 0.1017 | C |
| 105,545,147 | rs74354507 | *CBLB* | 3.87 | 4.48E-03 | 0.4237 | C | T | 0.0768 | T |
| 105,545,214 | rs12637560 | *CBLB* | 3.72 | 7.60E-03 | 0.3705 | C | T | 0.1016 | T |
| 105,547,637 | rs74739354 | *CBLB* | 3.88 | 4.46E-03 | 0.4242 | C | T | 0.0767 | T |
| 105,548,147 | rs78188796 | *CBLB* | 3.71 | 7.63E-03 | 0.3705 | T | C | 0.1016 | C |
| 105,558,837 | rs2028597 | *CBLB* | 3.88 | 4.34E-03 | 0.4260 | G | A | 0.0769 | A |
| 105,560,878 | rs78795990 | *CBLB* | 3.89 | 4.26E-03 | 0.4268 | G | A | 0.0769 | A |
| 105,567,334 | rs77127268 | *CBLB* | 3.89 | 4.27E-03 | 0.4216 | G | A | 0.0791 | A |
| 105,568,039 | rs76314485 | *CBLB* | 3.89 | 4.31E-03 | 0.4214 | C | T | 0.0791 | T |
| 105,576,057 | rs75471618 | *CBLB* | 3.85 | 4.81E-03 | 0.4187 | T | G | 0.0804 | G |
| 105,584,115 | rs75260404 | *CBLB* | 3.87 | 4.54E-03 | 0.4267 | G | A | 0.0767 | A |
| 105,602,251 | rs74615974 | *CBLB* | 3.92 | 3.83E-03 | 0.4309 | G | A | 0.0769 | A |
| 105,610,536 | rs75972220 | *CBLB* | 3.68 | 8.49E-03 | 0.3947 | T | C | 0.0844 | C |
| 105,611,338 | rs140985169 | *CBLB* | 3.77 | 6.34E-03 | 0.4309 | G | A | 0.0727 | A |
| 105,611,374 | rs150996865 | *CBLB* | 3.20 | 3.61E-02 | 0.3913 | G | T | 0.0628 | T |
| 105,627,263 | rs75925612 | *CBLB* | 3.71 | 7.76E-03 | 0.4256 | A | C | 0.0738 | C |
| 105,629,278 | rs80138759 | *CBLB* | 3.73 | 7.33E-03 | 0.4340 | G | T | 0.0695 | T |
| 105,640,704 | rs9870075 | *CBLB* | 3.51 | 1.48E-02 | 0.4048 | G | A | 0.0698 | A |
| 105,647,729 | rs77972468 | *CBLB* | 3.79 | 6.04E-03 | 0.4384 | T | G | 0.0728 | G |
| 106,212,808 | rs58222093 | *CBLB* | -3.14 | 4.27E-02 | -0.3612 | T | C | 0.0688 | T |
| 106,216,741 | rs79340394 | *CBLB* | -3.22 | 3.47E-02 | -0.3044 | A | C | 0.1044 | A |
| 107,307,503 | rs1697692 | *CD47* | -3.44 | 1.81E-02 | -0.2060 | C | T | 0.5383 | C |
| 107,313,250 | rs696377 | *CD47* | 3.46 | 1.70E-02 | 0.2061 | G | T | 0.4601 | T |
| 107,326,908 | rs602959 | *CD47* | 3.72 | 7.47E-03 | 0.2204 | C | T | 0.4698 | T |
| 107,386,894 | rs329923 | *CD47* | 3.45 | 1.73E-02 | 0.2080 | G | A | 0.3961 | A |
| 107,387,116 | rs329922 | *CD47* | 3.48 | 1.61E-02 | 0.2100 | G | A | 0.3959 | A |
| 107,545,050 | rs9832537 | *CD47* | 3.58 | 1.18E-02 | 0.2304 | G | C | 0.3319 | C |
| 107,550,819 | rs1908324 | *CD47* | 3.83 | 5.29E-03 | 0.2512 | C | T | 0.3122 | T |
| 107,597,927 | rs6776460 | *CD47* | 3.37 | 2.23E-02 | 0.3058 | A | G | 0.8568 | G |
| 107,604,277 | rs840286 | *CD47* | 3.59 | 1.14E-02 | 0.2399 | C | T | 0.7337 | T |
| 107,609,481 | rs709524 | *CD47* | 3.59 | 1.13E-02 | 0.2246 | G | A | 0.6249 | A |
| 107,609,990 | rs709523 | *CD47* | 3.59 | 1.14E-02 | 0.2243 | C | T | 0.6251 | T |
| 107,610,473 | rs709522 | *CD47* | 3.59 | 1.14E-02 | 0.2241 | A | G | 0.6249 | G |
| 107,611,941 | rs709521 | *CD47* | 3.55 | 1.30E-02 | 0.2207 | C | T | 0.6251 | T |
| 107,613,923 | rs709520 | *CD47* | 3.55 | 1.31E-02 | 0.2206 | T | C | 0.6248 | C |
| 107,617,229 | rs840288 | *CD47* | 3.55 | 1.27E-02 | 0.2213 | A | G | 0.6236 | G |
| 107,617,663 | rs709518 | *CD47* | 3.54 | 1.31E-02 | 0.2205 | T | C | 0.6251 | C |
| 107,618,514 | rs709517 | *CD47* | 3.54 | 1.31E-02 | 0.2205 | T | C | 0.6251 | C |
| 107,620,659 | rs709515 | *CD47* | 3.32 | 2.57E-02 | 0.2099 | T | C | 0.6947 | C |
| 107,620,896 | rs709514 | *CD47* | 3.37 | 2.24E-02 | 0.2096 | G | A | 0.6262 | A |
| 107,624,530 | rs4247450 | *CD47* | 3.31 | 2.69E-02 | 0.2091 | A | C | 0.6945 | C |
| 107,625,207 | rs11707678 | *CD47* | 3.38 | 2.17E-02 | 0.2103 | A | G | 0.6247 | G |
| 107,626,777 | rs1700452 | *CD47* | 3.37 | 2.24E-02 | 0.2096 | A | C | 0.6261 | C |
| 107,626,808 | rs1432595 | *CD47* | 3.37 | 2.22E-02 | 0.2098 | A | G | 0.6260 | G |
| 107,627,378 | rs1437235 | *CD47* | 3.37 | 2.22E-02 | 0.2098 | C | G | 0.6260 | G |
| 107,627,532 | rs1437234 | *CD47* | 3.37 | 2.24E-02 | 0.2096 | C | T | 0.6260 | T |
| 107,627,569 | rs1437233 | *CD47* | 3.37 | 2.24E-02 | 0.2097 | C | T | 0.6258 | T |
| 107,627,644 | rs1437232 | *CD47* | 3.37 | 2.24E-02 | 0.2097 | C | T | 0.6260 | T |
| 107,628,526 | rs1653906 | *CD47* | 3.37 | 2.26E-02 | 0.2096 | A | G | 0.6262 | G |
| 107,629,427 | rs1700456 | *CD47* | 3.37 | 2.26E-02 | 0.2097 | C | T | 0.6259 | T |
| 107,629,549 | rs1653907 | *CD47* | 3.37 | 2.26E-02 | 0.2097 | A | C | 0.6259 | C |
| 107,629,975 | rs4974375 | *CD47* | 3.37 | 2.21E-02 | 0.2103 | C | G | 0.6268 | G |
| 107,629,982 | rs4974374 | *CD47* | 3.37 | 2.21E-02 | 0.2103 | G | C | 0.6265 | C |
| 107,630,178 | rs1700457 | *CD47* | 3.37 | 2.25E-02 | 0.2098 | T | C | 0.6261 | C |
| 107,630,289 | rs1347651 | *CD47* | 3.37 | 2.25E-02 | 0.2098 | G | C | 0.6261 | C |
| 107,630,310 | rs1370283 | *CD47* | 3.36 | 2.27E-02 | 0.2096 | T | G | 0.6261 | G |
| 107,630,420 | rs1368255 | *CD47* | 3.37 | 2.25E-02 | 0.2098 | C | T | 0.6261 | T |
| 107,763,040 | rs2271054 | *CD47* | -3.12 | 4.44E-02 | -0.2456 | G | A | 0.1605 | G |
| 107,765,249 | rs3206652 | *CD47* | 3.53 | 1.36E-02 | 0.2266 | T | C | 0.2800 | C |
| 107,777,051 | rs3762679 | *CD47* | 3.60 | 1.11E-02 | 0.2306 | C | A | 0.2800 | A |
| 107,780,738 | rs3804638 | *CD47* | 3.62 | 1.04E-02 | 0.2316 | T | C | 0.2800 | C |
| 107,789,790 | rs56063635 | *CD47* | 3.52 | 1.43E-02 | 0.2606 | T | A | 0.1919 | A |
| 107,790,835 | rs3804639 | *CD47* | 3.60 | 1.09E-02 | 0.2314 | G | T | 0.2802 | T |
| 107,795,372 | rs12638945 | *CD47* | 3.72 | 7.58E-03 | 0.2391 | C | T | 0.2793 | T |
| 107,803,503 | rs35397021 | *CD47* | -3.15 | 4.14E-02 | -0.2474 | A | C | 0.1658 | A |
| 107,879,986 | rs428321 | *CD47* | -3.40 | 2.05E-02 | -0.3847 | T | C | 0.9323 | T |
| 107,888,360 | rs327164 | *CD47* | -3.40 | 2.05E-02 | -0.3847 | A | G | 0.9323 | A |
| 108,051,504 | rs17241659 | *CD47* | 3.35 | 2.34E-02 | 0.2360 | G | A | 0.2330 | A |
| 107,542,605 | rs2131949 | *IFT57* | 3.44 | 1.82E-02 | 0.2421 | G | T | 0.2428 | T |
| 107,818,777 | rs7635063 | *IFT57* | -3.15 | 4.18E-02 | -0.1972 | G | A | 0.3305 | G |
| 107,820,063 | rs7640424 | *IFT57* | -4.18 | 1.53E-03 | -0.2668 | C | T | 0.3098 | C |
| 107,820,388 | rs326360 | *IFT57* | 4.38 | 7.07E-04 | 0.3661 | G | A | 0.1432 | A |
| 107,821,546 | rs17828045 | *IFT57* | -4.28 | 1.04E-03 | -0.2647 | T | G | 0.3405 | T |
| 107,822,245 | rs1561029 | *IFT57* | 4.78 | 1.32E-04 | 0.2853 | A | C | 0.6241 | C |
| 107,822,310 | rs326358 | *IFT57* | 4.27 | 1.07E-03 | 0.2641 | C | T | 0.6589 | T |
| 107,822,481 | rs327139 | *IFT57* | 4.19 | 1.47E-03 | 0.2598 | G | C | 0.6606 | C |
| 107,824,061 | rs162064 | *IFT57* | 4.80 | 1.26E-04 | 0.2861 | T | G | 0.6415 | G |
| 107,824,222 | rs34154497 | *IFT57* | -4.31 | 9.35E-04 | -0.2679 | T | C | 0.3236 | T |
| 107,824,563 | rs326356 | *IFT57* | 4.87 | 9.24E-05 | 0.2900 | A | G | 0.6467 | G |
| 107,824,663 | rs326355 | *IFT57* | 4.87 | 9.12E-05 | 0.2901 | T | C | 0.6467 | C |
| 107,828,001 | rs4855619 | *IFT57* | 6.25 | 9.06E-08 | 0.5909 | C | T | 0.1066 | T |
| 107,828,479 | rs7649316 | *IFT57* | 6.25 | 9.09E-08 | 0.5908 | C | T | 0.1066 | T |
| 107,829,484 | rs34937412 | *IFT57* | -4.55 | 3.51E-04 | -0.2798 | G | C | 0.3219 | G |
| 107,829,507 | rs35168062 | *IFT57* | -5.41 | 7.69E-06 | -0.3169 | A | G | 0.3642 | A |
| 107,829,562 | rs17231712 | *IFT57* | -5.33 | 1.10E-05 | -0.3126 | C | G | 0.3632 | C |
| 107,830,164 | rs13075747 | *IFT57* | -5.41 | 7.58E-06 | -0.3172 | T | A | 0.3642 | T |
| 107,830,245 | rs12490871 | *IFT57* | 6.24 | 9.24E-08 | 0.5905 | A | G | 0.1066 | G |
| 107,832,768 | rs62264093 | *IFT57* | 6.24 | 9.24E-08 | 0.5905 | G | A | 0.1065 | A |
| 107,833,289 | rs7648181 | *IFT57* | 5.38 | 8.72E-06 | 0.3155 | C | A | 0.6359 | A |
| 107,835,448 | rs162070 | *IFT57* | 4.53 | 3.81E-04 | 0.2785 | G | A | 0.6787 | A |
| 107,838,805 | rs161880 | *IFT57* | 5.26 | 1.58E-05 | 0.3101 | T | C | 0.6394 | C |
| 107,838,891 | rs182736 | *IFT57* | 4.46 | 5.09E-04 | 0.2741 | C | A | 0.6798 | A |
| 107,839,341 | rs162069 | *IFT57* | 5.26 | 1.57E-05 | 0.3102 | A | G | 0.6394 | G |
| 107,840,773 | rs179046 | *IFT57* | 4.45 | 5.22E-04 | 0.2738 | G | T | 0.6801 | T |
| 107,840,830 | rs170534 | *IFT57* | 5.32 | 1.18E-05 | 0.3129 | T | C | 0.6378 | C |
| 107,841,077 | rs62264095 | *IFT57* | 6.12 | 1.83E-07 | 0.5763 | T | C | 0.1077 | C |
| 107,843,004 | rs326338 | *IFT57* | 5.39 | 8.52E-06 | 0.3166 | T | C | 0.6381 | C |
| 107,844,017 | rs11926884 | *IFT57* | 6.12 | 1.83E-07 | 0.5763 | T | C | 0.1079 | C |
| 107,845,395 | rs68044584 | *IFT57* | -4.62 | 2.63E-04 | -0.2803 | A | G | 0.3338 | A |
| 107,845,553 | rs11922336 | *IFT57* | 6.76 | 4.51E-09 | 0.6672 | T | A | 0.0968 | A |
| 107,846,617 | rs17232205 | *IFT57* | 6.71 | 6.09E-09 | 0.6538 | C | A | 0.1001 | A |
| 107,847,696 | rs62264097 | *IFT57* | 6.71 | 6.09E-09 | 0.6538 | G | A | 0.1001 | A |
| 107,847,725 | rs9839167 | *IFT57* | 6.71 | 6.09E-09 | 0.6538 | G | A | 0.1006 | A |
| 107,847,902 | rs7633919 | *IFT57* | 6.71 | 6.09E-09 | 0.6538 | C | A | 0.1006 | A |
| 107,851,467 | rs13074183 | *IFT57* | -4.93 | 6.99E-05 | -0.2977 | G | A | 0.3302 | G |
| 107,859,558 | rs35703078 | *IFT57* | -4.98 | 5.56E-05 | -0.3030 | A | G | 0.3275 | A |
| 107,861,766 | rs12152307 | *IFT57* | -4.97 | 5.79E-05 | -0.3026 | T | C | 0.3276 | T |
| 107,864,673 | rs11917909 | *IFT57* | -4.89 | 8.53E-05 | -0.2925 | G | A | 0.3541 | G |
| 107,864,963 | rs13093976 | *IFT57* | -4.88 | 8.59E-05 | -0.2924 | G | T | 0.3541 | G |
| 107,865,263 | rs9822398 | *IFT57* | 6.69 | 6.88E-09 | 0.6493 | T | C | 0.1005 | C |
| 107,865,763 | rs10514749 | *IFT57* | 6.69 | 6.90E-09 | 0.6493 | C | T | 0.1005 | T |
| 107,866,326 | rs57645341 | *IFT57* | 6.69 | 6.88E-09 | 0.6493 | G | A | 0.1004 | A |
| 107,867,826 | rs57182717 | *IFT57* | 6.69 | 6.88E-09 | 0.6493 | C | T | 0.1004 | T |
| 107,868,027 | rs57585440 | *IFT57* | 6.69 | 6.88E-09 | 0.6493 | G | A | 0.1004 | A |
| 107,868,110 | rs59107359 | *IFT57* | 6.69 | 6.94E-09 | 0.6490 | T | C | 0.1004 | C |
| 107,868,272 | rs60140727 | *IFT57* | 6.69 | 6.88E-09 | 0.6493 | A | G | 0.1004 | G |
| 107,868,442 | rs13069689 | *IFT57* | -4.00 | 2.85E-03 | -0.2698 | C | T | 0.2421 | C |
| 107,868,661 | rs10514750 | *IFT57* | 6.69 | 6.88E-09 | 0.6493 | G | A | 0.1004 | A |
| 107,869,757 | rs62264106 | *IFT57* | 6.90 | 2.02E-09 | 0.6698 | A | C | 0.1002 | C |
| 107,869,895 | rs17232450 | *IFT57* | 6.90 | 1.91E-09 | 0.6706 | C | T | 0.1002 | T |
| 107,870,139 | rs2117761 | *IFT57* | 6.90 | 1.91E-09 | 0.6706 | A | T | 0.1002 | T |
| 107,870,306 | rs60080056 | *IFT57* | 6.90 | 1.91E-09 | 0.6706 | T | C | 0.1002 | C |
| 107,870,835 | rs13082569 | *IFT57* | -4.88 | 8.59E-05 | -0.2924 | C | A | 0.3540 | C |
| 107,871,847 | rs58300903 | *IFT57* | 6.82 | 3.28E-09 | 0.6833 | A | G | 0.0979 | G |
| 107,871,854 | rs11920542 | *IFT57* | 6.81 | 3.30E-09 | 0.6834 | C | T | 0.0979 | T |
| 107,871,870 | rs57414689 | *IFT57* | 6.80 | 3.54E-09 | 0.6810 | C | T | 0.0984 | T |
| 107,871,891 | rs58004035 | *IFT57* | 6.78 | 4.00E-09 | 0.6775 | C | A | 0.0988 | A |
| 107,872,665 | rs11920797 | *IFT57* | 6.90 | 1.91E-09 | 0.6706 | C | T | 0.1001 | T |
| 107,872,922 | rs11921625 | *IFT57* | 6.90 | 1.91E-09 | 0.6706 | C | A | 0.1001 | A |
| 107,873,527 | rs62264141 | *IFT57* | 6.90 | 1.90E-09 | 0.6706 | T | A | 0.1001 | A |
| 107,874,638 | rs13063741 | *IFT57* | -4.88 | 8.79E-05 | -0.2919 | C | T | 0.3535 | C |
| 107,875,733 | rs62264142 | *IFT57* | 6.91 | 1.90E-09 | 0.6707 | T | C | 0.1001 | C |
| 107,876,232 | rs6795280 | *IFT57* | -4.88 | 8.77E-05 | -0.2919 | T | C | 0.3523 | T |
| 107,876,247 | rs34161295 | *IFT57* | 6.90 | 1.91E-09 | 0.6707 | T | C | 0.1001 | C |
| 107,876,367 | rs11926978 | *IFT57* | 6.91 | 1.89E-09 | 0.6707 | A | G | 0.1001 | G |
| 107,876,432 | rs11919348 | *IFT57* | 6.91 | 1.89E-09 | 0.6707 | G | A | 0.1002 | A |
| 107,876,697 | rs62264143 | *IFT57* | 6.91 | 1.89E-09 | 0.6707 | T | C | 0.1000 | C |
| 107,876,850 | rs11923518 | *IFT57* | 6.91 | 1.89E-09 | 0.6707 | T | G | 0.1000 | G |
| 107,877,134 | rs13091474 | *IFT57* | -4.88 | 8.78E-05 | -0.2919 | A | T | 0.3522 | A |
| 107,877,463 | rs17232556 | *IFT57* | 6.91 | 1.89E-09 | 0.6707 | C | T | 0.1000 | T |
| 107,877,538 | rs17232563 | *IFT57* | 6.91 | 1.89E-09 | 0.6707 | G | C | 0.1000 | C |
| 107,878,213 | rs76893212 | *IFT57* | 6.91 | 1.89E-09 | 0.6707 | C | T | 0.1000 | T |
| 107,879,274 | rs62264144 | *IFT57* | 6.91 | 1.89E-09 | 0.6707 | T | C | 0.1000 | C |
| 107,879,516 | rs62264145 | *IFT57* | 6.91 | 1.89E-09 | 0.6707 | T | C | 0.1000 | C |
| 107,879,730 | rs62264146 | *IFT57* | 6.91 | 1.89E-09 | 0.6707 | G | C | 0.1000 | C |
| 107,880,473 | rs17828926 | *IFT57* | 6.91 | 1.89E-09 | 0.6707 | T | G | 0.1000 | G |
| 107,880,919 | rs2035 | *IFT57* | 6.82 | 3.25E-09 | 0.6543 | T | C | 0.1005 | C |
| 107,880,977 | rs57535746 | *IFT57* | 6.91 | 1.89E-09 | 0.6708 | G | A | 0.1000 | A |
| 107,881,138 | rs1135937 | *IFT57* | 6.91 | 1.89E-09 | 0.6708 | T | A | 0.1000 | A |
| 107,881,152 | rs58273586 | *IFT57* | 6.91 | 1.89E-09 | 0.6708 | T | C | 0.1000 | C |
| 107,881,701 | rs9860418 | *IFT57* | 6.91 | 1.89E-09 | 0.6708 | G | A | 0.1000 | A |
| 107,881,711 | rs62264147 | *IFT57* | 6.91 | 1.89E-09 | 0.6708 | T | C | 0.1000 | C |
| 107,881,796 | rs9860587 | *IFT57* | 6.91 | 1.89E-09 | 0.6708 | G | A | 0.1000 | A |
| 107,882,291 | rs28494587 | *IFT57* | 6.91 | 1.89E-09 | 0.6708 | G | T | 0.1000 | T |
| 107,882,357 | rs28483736 | *IFT57* | 6.91 | 1.89E-09 | 0.6708 | C | T | 0.1000 | T |
| 107,882,952 | rs79163761 | *IFT57* | 6.91 | 1.89E-09 | 0.6708 | T | C | 0.1000 | C |
| 107,883,232 | rs9814678 | *IFT57* | 6.91 | 1.89E-09 | 0.6708 | T | C | 0.1000 | C |
| 107,883,530 | rs13325340 | *IFT57* | 6.91 | 1.89E-09 | 0.6708 | C | G | 0.1000 | G |
| 107,883,768 | rs13318436 | *IFT57* | 6.91 | 1.89E-09 | 0.6708 | A | G | 0.1000 | G |
| 107,884,132 | rs62264149 | *IFT57* | 6.91 | 1.89E-09 | 0.6708 | T | C | 0.1000 | C |
| 107,884,269 | rs62264150 | *IFT57* | 6.91 | 1.89E-09 | 0.6708 | A | G | 0.1000 | G |
| 107,884,825 | rs9838360 | *IFT57* | 6.91 | 1.89E-09 | 0.6708 | A | C | 0.1000 | C |
| 107,884,928 | rs9814209 | *IFT57* | 6.91 | 1.89E-09 | 0.6708 | A | G | 0.1000 | G |
| 107,885,076 | rs62264151 | *IFT57* | 6.91 | 1.89E-09 | 0.6708 | C | T | 0.1000 | T |
| 107,885,105 | rs9852283 | *IFT57* | 6.91 | 1.89E-09 | 0.6708 | C | T | 0.1000 | T |
| 107,885,483 | rs17232794 | *IFT57* | 6.91 | 1.89E-09 | 0.6708 | G | C | 0.1000 | C |
| 107,885,875 | rs2305551 | *IFT57* | 6.91 | 1.89E-09 | 0.6708 | A | G | 0.1000 | G |
| 107,886,925 | rs17232836 | *IFT57* | 6.91 | 1.89E-09 | 0.6708 | C | T | 0.1000 | T |
| 107,887,405 | rs72931629 | *IFT57* | 6.91 | 1.89E-09 | 0.6708 | C | T | 0.1000 | T |
| 107,887,756 | rs9826261 | *IFT57* | 6.91 | 1.89E-09 | 0.6708 | A | G | 0.1000 | G |
| 107,887,821 | rs62264152 | *IFT57* | 6.91 | 1.89E-09 | 0.6708 | C | T | 0.1000 | T |
| 107,887,906 | rs9830118 | *IFT57* | 6.91 | 1.89E-09 | 0.6708 | A | C | 0.1000 | C |
| 107,887,934 | rs62264153 | *IFT57* | 6.91 | 1.89E-09 | 0.6708 | T | G | 0.1000 | G |
| 107,888,248 | rs34992087 | *IFT57* | 6.91 | 1.89E-09 | 0.6708 | A | G | 0.1000 | G |
| 107,888,443 | rs36046592 | *IFT57* | 6.91 | 1.89E-09 | 0.6708 | C | T | 0.1000 | T |
| 107,888,523 | rs13083896 | *IFT57* | 6.91 | 1.89E-09 | 0.6708 | A | T | 0.1000 | T |
| 107,888,757 | rs9869274 | *IFT57* | 6.91 | 1.89E-09 | 0.6708 | C | T | 0.1000 | T |
| 107,888,841 | rs36131051 | *IFT57* | -3.49 | 1.55E-02 | -0.2660 | T | G | 0.2086 | T |
| 107,889,142 | rs140568382 | *IFT57* | 6.91 | 1.89E-09 | 0.6707 | G | A | 0.1000 | A |
| 107,889,747 | rs9874789 | *IFT57* | 6.91 | 1.89E-09 | 0.6707 | G | T | 0.1000 | T |
| 107,889,963 | rs9823406 | *IFT57* | 6.91 | 1.89E-09 | 0.6707 | T | C | 0.1000 | C |
| 107,890,061 | rs11928732 | *IFT57* | 6.91 | 1.89E-09 | 0.6707 | G | A | 0.1000 | A |
| 107,890,218 | rs11928715 | *IFT57* | 6.91 | 1.90E-09 | 0.6707 | C | T | 0.1000 | T |
| 107,890,304 | rs11917405 | *IFT57* | 6.91 | 1.89E-09 | 0.6707 | T | C | 0.1000 | C |
| 107,890,460 | rs11921090 | *IFT57* | 6.91 | 1.89E-09 | 0.6707 | A | G | 0.1000 | G |
| 107,890,927 | rs17829213 | *IFT57* | 6.91 | 1.89E-09 | 0.6707 | T | C | 0.1000 | C |
| 107,891,065 | rs17829242 | *IFT57* | 6.91 | 1.89E-09 | 0.6707 | C | T | 0.1000 | T |
| 107,891,196 | rs28369138 | *IFT57* | 6.91 | 1.89E-09 | 0.6707 | A | G | 0.1000 | G |
| 107,891,213 | rs28599946 | *IFT57* | 6.91 | 1.89E-09 | 0.6707 | A | G | 0.1000 | G |
| 107,891,451 | rs17232997 | *IFT57* | 6.91 | 1.89E-09 | 0.6707 | A | G | 0.1000 | G |
| 107,892,781 | rs59499415 | *IFT57* | 6.91 | 1.89E-09 | 0.6707 | T | C | 0.1000 | C |
| 107,893,058 | rs58006907 | *IFT57* | 6.91 | 1.89E-09 | 0.6707 | A | G | 0.1000 | G |
| 107,893,149 | rs10514751 | *IFT57* | 6.53 | 1.76E-08 | 0.6528 | T | C | 0.0970 | C |
| 107,893,428 | rs28406754 | *IFT57* | 6.91 | 1.89E-09 | 0.6707 | T | G | 0.1000 | G |
| 107,893,579 | rs28377152 | *IFT57* | 6.91 | 1.89E-09 | 0.6707 | T | C | 0.1000 | C |
| 107,893,637 | rs28522908 | *IFT57* | 6.91 | 1.89E-09 | 0.6707 | C | G | 0.1000 | G |
| 107,893,740 | rs62262369 | *IFT57* | 6.91 | 1.89E-09 | 0.6707 | A | T | 0.1000 | T |
| 107,893,972 | rs9825043 | *IFT57* | 6.91 | 1.89E-09 | 0.6707 | G | A | 0.1000 | A |
| 107,894,104 | rs9844904 | *IFT57* | 6.91 | 1.89E-09 | 0.6707 | T | A | 0.1000 | A |
| 107,894,117 | rs9844911 | *IFT57* | 6.91 | 1.89E-09 | 0.6707 | T | C | 0.1000 | C |
| 107,894,492 | rs11917750 | *IFT57* | 6.91 | 1.89E-09 | 0.6707 | C | A | 0.1000 | A |
| 107,894,592 | rs723271 | *IFT57* | 6.91 | 1.89E-09 | 0.6707 | T | C | 0.1000 | C |
| 107,894,840 | rs723272 | *IFT57* | 6.91 | 1.89E-09 | 0.6707 | C | A | 0.1000 | A |
| 107,894,989 | rs723273 | *IFT57* | 6.91 | 1.89E-09 | 0.6708 | G | A | 0.1000 | A |
| 107,895,038 | rs1369554 | *IFT57* | 6.91 | 1.89E-09 | 0.6707 | T | A | 0.1000 | A |
| 107,895,207 | rs7610003 | *IFT57* | 6.91 | 1.89E-09 | 0.6707 | C | T | 0.1000 | T |
| 107,895,326 | rs7632036 | *IFT57* | 6.91 | 1.89E-09 | 0.6707 | A | G | 0.1000 | G |
| 107,895,496 | rs9822625 | *IFT57* | 6.91 | 1.89E-09 | 0.6707 | T | C | 0.1000 | C |
| 107,895,579 | rs11917179 | *IFT57* | 6.91 | 1.89E-09 | 0.6707 | T | C | 0.1000 | C |
| 107,895,593 | rs9878123 | *IFT57* | 6.91 | 1.89E-09 | 0.6707 | C | T | 0.1000 | T |
| 107,895,734 | rs9822968 | *IFT57* | 6.91 | 1.89E-09 | 0.6707 | T | C | 0.1000 | C |
| 107,895,761 | rs9840611 | *IFT57* | 6.91 | 1.89E-09 | 0.6707 | A | C | 0.1000 | C |
| 107,895,891 | rs9826950 | *IFT57* | 6.91 | 1.89E-09 | 0.6707 | T | C | 0.1000 | C |
| 107,896,075 | rs62262370 | *IFT57* | 6.91 | 1.89E-09 | 0.6707 | T | C | 0.1000 | C |
| 107,896,108 | rs62262371 | *IFT57* | -4.88 | 8.79E-05 | -0.2919 | C | T | 0.3521 | C |
| 107,896,171 | rs1982435 | *IFT57* | 6.90 | 1.90E-09 | 0.6706 | T | C | 0.1001 | C |
| 107,896,562 | rs62262372 | *IFT57* | 6.91 | 1.89E-09 | 0.6707 | T | C | 0.1000 | C |
| 107,896,711 | rs9883627 | *IFT57* | 6.91 | 1.89E-09 | 0.6707 | C | G | 0.1000 | G |
| 107,896,972 | rs62262373 | *IFT57* | 6.91 | 1.89E-09 | 0.6707 | A | C | 0.1000 | C |
| 107,896,984 | rs11919185 | *IFT57* | 6.91 | 1.89E-09 | 0.6707 | T | C | 0.1000 | C |
| 107,897,239 | rs11922835 | *IFT57* | 6.91 | 1.89E-09 | 0.6707 | A | G | 0.1005 | G |
| 107,897,294 | rs11915212 | *IFT57* | 6.91 | 1.89E-09 | 0.6707 | G | A | 0.1000 | A |
| 107,897,545 | rs11915231 | *IFT57* | 6.91 | 1.89E-09 | 0.6707 | C | A | 0.1000 | A |
| 107,897,654 | rs62262374 | *IFT57* | 6.91 | 1.89E-09 | 0.6707 | T | A | 0.1000 | A |
| 107,897,706 | rs62262375 | *IFT57* | 6.91 | 1.89E-09 | 0.6707 | C | T | 0.1000 | T |
| 107,898,136 | rs17233199 | *IFT57* | 6.91 | 1.89E-09 | 0.6707 | G | A | 0.1000 | A |
| 107,898,593 | rs9818755 | *IFT57* | 6.91 | 1.89E-09 | 0.6707 | C | T | 0.1000 | T |
| 107,898,727 | rs9818915 | *IFT57* | 6.91 | 1.89E-09 | 0.6707 | C | T | 0.1000 | T |
| 107,899,338 | rs17829434 | *IFT57* | 6.91 | 1.89E-09 | 0.6707 | C | A | 0.1000 | A |
| 107,899,513 | rs17233248 | *IFT57* | -4.88 | 8.76E-05 | -0.2919 | T | C | 0.3521 | T |
| 107,899,517 | rs62262376 | *IFT57* | 6.91 | 1.89E-09 | 0.6707 | C | T | 0.1000 | T |
| 107,899,580 | rs7634546 | *IFT57* | 6.91 | 1.89E-09 | 0.6707 | T | C | 0.1000 | C |
| 107,899,658 | rs7622938 | *IFT57* | 6.91 | 1.89E-09 | 0.6707 | C | T | 0.1000 | T |
| 107,899,961 | rs7647452 | *IFT57* | 6.91 | 1.89E-09 | 0.6707 | A | T | 0.1000 | T |
| 107,900,102 | rs35444506 | *IFT57* | 6.91 | 1.89E-09 | 0.6707 | A | G | 0.1000 | G |
| 107,900,451 | rs62262378 | *IFT57* | 6.91 | 1.89E-09 | 0.6707 | T | C | 0.1000 | C |
| 107,900,739 | rs9829875 | *IFT57* | 6.91 | 1.89E-09 | 0.6707 | G | A | 0.1000 | A |
| 107,901,065 | rs17829536 | *IFT57* | 6.91 | 1.89E-09 | 0.6707 | G | A | 0.1000 | A |
| 107,901,184 | rs1369555 | *IFT57* | -4.88 | 8.77E-05 | -0.2919 | G | A | 0.3526 | G |
| 107,901,222 | rs72931696 | *IFT57* | 6.91 | 1.89E-09 | 0.6707 | T | C | 0.1000 | C |
| 107,901,605 | rs62262379 | *IFT57* | 6.91 | 1.89E-09 | 0.6707 | C | A | 0.1000 | A |
| 107,901,643 | rs62262380 | *IFT57* | 6.91 | 1.89E-09 | 0.6707 | T | C | 0.1000 | C |
| 107,901,822 | rs17829572 | *IFT57* | 6.91 | 1.89E-09 | 0.6707 | G | C | 0.1000 | C |
| 107,902,059 | rs62262381 | *IFT57* | 6.91 | 1.89E-09 | 0.6707 | C | T | 0.1000 | T |
| 107,902,083 | rs10511268 | *IFT57* | 6.91 | 1.89E-09 | 0.6707 | C | T | 0.1000 | T |
| 107,902,228 | rs62262382 | *IFT57* | 6.91 | 1.89E-09 | 0.6707 | C | A | 0.1000 | A |
| 107,902,464 | rs13323383 | *IFT57* | 6.91 | 1.89E-09 | 0.6707 | T | C | 0.1000 | C |
| 107,902,512 | rs13326673 | *IFT57* | 6.91 | 1.89E-09 | 0.6707 | A | T | 0.1000 | T |
| 107,902,593 | rs13323413 | *IFT57* | 6.91 | 1.89E-09 | 0.6707 | T | A | 0.1000 | A |
| 107,902,883 | rs62262383 | *IFT57* | 6.91 | 1.89E-09 | 0.6707 | A | G | 0.1000 | G |
| 107,903,001 | rs10511269 | *IFT57* | 6.91 | 1.89E-09 | 0.6707 | G | A | 0.1000 | A |
| 107,903,075 | rs10514752 | *IFT57* | 6.91 | 1.89E-09 | 0.6707 | A | C | 0.1000 | C |
| 107,903,216 | rs68147515 | *IFT57* | -4.88 | 8.77E-05 | -0.2919 | T | A | 0.3525 | T |
| 107,903,486 | rs62262413 | *IFT57* | 6.91 | 1.89E-09 | 0.6707 | A | G | 0.1000 | G |
| 107,903,581 | rs62262414 | *IFT57* | 6.91 | 1.89E-09 | 0.6707 | T | C | 0.1000 | C |
| 107,903,788 | rs62262415 | *IFT57* | 6.91 | 1.89E-09 | 0.6707 | T | C | 0.1000 | C |
| 107,904,047 | rs72933708 | *IFT57* | 6.91 | 1.89E-09 | 0.6707 | A | G | 0.1000 | G |
| 107,904,070 | rs72933710 | *IFT57* | 6.91 | 1.89E-09 | 0.6707 | A | G | 0.1000 | G |
| 107,904,438 | rs9850721 | *IFT57* | 6.91 | 1.89E-09 | 0.6707 | G | A | 0.1000 | A |
| 107,905,339 | rs13433942 | *IFT57* | 6.91 | 1.89E-09 | 0.6707 | A | G | 0.1000 | G |
| 107,905,381 | rs57119311 | *IFT57* | 6.91 | 1.89E-09 | 0.6707 | G | A | 0.1000 | A |
| 107,905,760 | rs9839306 | *IFT57* | 6.91 | 1.89E-09 | 0.6707 | A | C | 0.1000 | C |
| 107,906,242 | rs9881619 | *IFT57* | 6.91 | 1.89E-09 | 0.6707 | C | T | 0.1000 | T |
| 107,906,638 | rs17239910 | *IFT57* | 6.91 | 1.89E-09 | 0.6707 | A | G | 0.1000 | G |
| 107,907,175 | rs62262418 | *IFT57* | 6.91 | 1.89E-09 | 0.6707 | T | C | 0.1000 | C |
| 107,907,526 | rs9832007 | *IFT57* | 6.91 | 1.89E-09 | 0.6707 | T | C | 0.1000 | C |
| 107,907,818 | rs9288861 | *IFT57* | 6.91 | 1.89E-09 | 0.6707 | T | C | 0.1000 | C |
| 107,907,863 | rs9853929 | *IFT57* | 6.91 | 1.89E-09 | 0.6707 | A | C | 0.1000 | C |
| 107,907,924 | rs9816413 | *IFT57* | 6.91 | 1.89E-09 | 0.6707 | C | T | 0.1000 | T |
| 107,907,974 | rs9288862 | *IFT57* | 6.91 | 1.89E-09 | 0.6707 | T | C | 0.1000 | C |
| 107,908,000 | rs9836408 | *IFT57* | 6.91 | 1.89E-09 | 0.6707 | T | C | 0.1000 | C |
| 107,908,181 | rs17239988 | *IFT57* | 6.91 | 1.89E-09 | 0.6707 | C | T | 0.1000 | T |
| 107,908,391 | rs9854728 | *IFT57* | 6.91 | 1.89E-09 | 0.6707 | A | G | 0.1000 | G |
| 107,909,034 | rs9841882 | *IFT57* | 6.91 | 1.89E-09 | 0.6707 | T | C | 0.1000 | C |
| 107,909,249 | rs9859807 | *IFT57* | 6.91 | 1.89E-09 | 0.6708 | A | C | 0.1000 | C |
| 107,909,299 | rs9859939 | *IFT57* | 6.91 | 1.89E-09 | 0.6708 | A | G | 0.1000 | G |
| 107,909,788 | rs12152366 | *IFT57* | 6.91 | 1.89E-09 | 0.6708 | T | C | 0.1000 | C |
| 107,909,940 | rs62262420 | *IFT57* | -4.85 | 1.01E-04 | -0.2903 | C | T | 0.3521 | C |
| 107,911,183 | rs9832812 | *IFT57* | 6.91 | 1.89E-09 | 0.6709 | C | T | 0.1000 | T |
| 107,911,198 | rs9832818 | *IFT57* | 6.91 | 1.89E-09 | 0.6709 | C | T | 0.1000 | T |
| 107,911,523 | rs9853355 | *IFT57* | 6.91 | 1.89E-09 | 0.6709 | T | G | 0.0999 | G |
| 107,911,545 | rs9853363 | *IFT57* | 6.91 | 1.89E-09 | 0.6709 | T | A | 0.0999 | A |
| 107,911,608 | rs9833455 | *IFT57* | 6.91 | 1.89E-09 | 0.6709 | C | T | 0.0999 | T |
| 107,911,670 | rs9871169 | *IFT57* | 6.91 | 1.89E-09 | 0.6709 | A | G | 0.0999 | G |
| 107,911,965 | rs12152431 | *IFT57* | 6.91 | 1.89E-09 | 0.6709 | T | C | 0.0999 | C |
| 107,912,208 | rs13319301 | *IFT57* | 6.91 | 1.89E-09 | 0.6710 | C | G | 0.0999 | G |
| 107,912,351 | rs13319331 | *IFT57* | 6.91 | 1.89E-09 | 0.6710 | C | T | 0.0999 | T |
| 107,912,435 | rs13323116 | *IFT57* | 6.91 | 1.89E-09 | 0.6710 | T | C | 0.0999 | C |
| 107,912,674 | rs62262421 | *IFT57* | 6.91 | 1.89E-09 | 0.6710 | T | A | 0.0999 | A |
| 107,913,059 | rs35710439 | *IFT57* | -4.87 | 9.14E-05 | -0.2915 | C | A | 0.3534 | C |
| 107,913,107 | rs1563042 | *IFT57* | 6.91 | 1.89E-09 | 0.6710 | A | T | 0.0999 | T |
| 107,913,849 | rs9882055 | *IFT57* | 6.91 | 1.89E-09 | 0.6711 | A | G | 0.0999 | G |
| 107,914,125 | rs57838153 | *IFT57* | 6.91 | 1.89E-09 | 0.6711 | C | T | 0.0999 | T |
| 107,914,145 | rs9848999 | *IFT57* | 6.91 | 1.89E-09 | 0.6711 | G | A | 0.0999 | A |
| 107,914,241 | rs9868740 | *IFT57* | 6.91 | 1.89E-09 | 0.6711 | T | C | 0.0999 | C |
| 107,914,313 | rs35798995 | *IFT57* | -4.88 | 8.77E-05 | -0.2919 | G | A | 0.3519 | G |
| 107,914,767 | rs11926812 | *IFT57* | 6.91 | 1.89E-09 | 0.6711 | T | G | 0.0999 | G |
| 107,915,197 | rs9863548 | *IFT57* | 6.91 | 1.89E-09 | 0.6712 | A | C | 0.0999 | C |
| 107,916,058 | rs13325007 | *IFT57* | 6.88 | 2.18E-09 | 0.6692 | A | G | 0.1000 | G |
| 107,916,126 | rs62267863 | *IFT57* | 6.91 | 1.89E-09 | 0.6712 | C | T | 0.0999 | T |
| 107,916,254 | rs13318118 | *IFT57* | 6.91 | 1.89E-09 | 0.6712 | C | A | 0.0999 | A |
| 107,916,266 | rs13318120 | *IFT57* | 6.91 | 1.89E-09 | 0.6712 | C | T | 0.0999 | T |
| 107,916,419 | rs62267864 | *IFT57* | 6.91 | 1.89E-09 | 0.6713 | T | C | 0.0999 | C |
| 107,916,925 | rs62267865 | *IFT57* | 6.91 | 1.89E-09 | 0.6713 | A | G | 0.0999 | G |
| 107,917,003 | rs9856316 | *IFT57* | 6.91 | 1.89E-09 | 0.6713 | T | G | 0.0999 | G |
| 107,917,232 | rs9836530 | *IFT57* | 6.91 | 1.89E-09 | 0.6713 | C | T | 0.0999 | T |
| 107,917,331 | rs62267866 | *IFT57* | 6.91 | 1.89E-09 | 0.6713 | C | G | 0.0999 | G |
| 107,917,824 | rs9857584 | *IFT57* | 6.91 | 1.89E-09 | 0.6714 | T | C | 0.0999 | C |
| 107,917,850 | rs9875102 | *IFT57* | 6.91 | 1.89E-09 | 0.6714 | A | G | 0.0999 | G |
| 107,918,641 | rs9862649 | *IFT57* | 6.89 | 2.13E-09 | 0.6699 | T | G | 0.0997 | G |
| 107,919,140 | rs72933753 | *IFT57* | 6.89 | 2.13E-09 | 0.6699 | T | C | 0.0997 | C |
| 107,919,291 | rs921582 | *IFT57* | 6.89 | 2.13E-09 | 0.6699 | G | A | 0.0997 | A |
| 107,919,348 | rs11926321 | *IFT57* | 6.89 | 2.13E-09 | 0.6699 | T | C | 0.0997 | C |
| 107,919,480 | rs921583 | *IFT57* | 6.89 | 2.13E-09 | 0.6699 | C | T | 0.0998 | T |
| 107,919,578 | rs4640571 | *IFT57* | 6.89 | 2.14E-09 | 0.6699 | A | G | 0.0997 | G |
| 107,919,930 | rs720021 | *IFT57* | 6.89 | 2.14E-09 | 0.6698 | C | T | 0.0997 | T |
| 107,920,003 | rs62267875 | *IFT57* | 6.89 | 2.14E-09 | 0.6698 | G | T | 0.0997 | T |
| 107,920,923 | rs9857746 | *IFT57* | 6.89 | 2.12E-09 | 0.6701 | G | A | 0.0997 | A |
| 107,920,938 | rs9877443 | *IFT57* | 6.89 | 2.14E-09 | 0.6699 | T | C | 0.0997 | C |
| 107,920,943 | rs9877444 | *IFT57* | 6.89 | 2.12E-09 | 0.6701 | T | C | 0.0997 | C |
| 107,920,979 | rs62267876 | *IFT57* | 6.89 | 2.15E-09 | 0.6698 | T | C | 0.0997 | C |
| 107,921,080 | rs1377275 | *IFT57* | 6.89 | 2.15E-09 | 0.6698 | G | A | 0.0997 | A |
| 107,921,088 | rs1349691 | *IFT57* | 6.89 | 2.15E-09 | 0.6698 | G | A | 0.0997 | A |
| 107,921,437 | rs9878221 | *IFT57* | 6.89 | 2.15E-09 | 0.6697 | T | C | 0.0997 | C |
| 107,921,855 | rs62267877 | *IFT57* | 6.88 | 2.15E-09 | 0.6697 | G | A | 0.0997 | A |
| 107,921,862 | rs9882728 | *IFT57* | 6.88 | 2.15E-09 | 0.6697 | T | C | 0.0997 | C |
| 107,922,116 | rs35595234 | *IFT57* | -4.88 | 8.73E-05 | -0.2921 | C | T | 0.3519 | C |
| 107,922,272 | rs9825709 | *IFT57* | 6.88 | 2.15E-09 | 0.6697 | A | C | 0.0997 | C |
| 107,922,571 | rs62267879 | *IFT57* | 6.88 | 2.16E-09 | 0.6697 | A | G | 0.0997 | G |
| 107,922,734 | rs9864340 | *IFT57* | 6.88 | 2.16E-09 | 0.6696 | G | T | 0.0997 | T |
| 107,923,251 | rs62267880 | *IFT57* | 6.88 | 2.16E-09 | 0.6696 | G | A | 0.0997 | A |
| 107,923,291 | rs9868639 | *IFT57* | 6.88 | 2.16E-09 | 0.6696 | C | G | 0.0998 | G |
| 107,923,740 | rs62267881 | *IFT57* | 6.88 | 2.16E-09 | 0.6696 | C | G | 0.0997 | G |
| 107,924,101 | rs9835895 | *IFT57* | 6.88 | 2.16E-09 | 0.6696 | A | G | 0.0998 | G |
| 107,924,233 | rs9836052 | *IFT57* | 6.88 | 2.16E-09 | 0.6696 | A | C | 0.0998 | C |
| 107,924,262 | rs9836172 | *IFT57* | 6.89 | 2.07E-09 | 0.6705 | A | G | 0.0997 | G |
| 107,925,208 | rs9870968 | *IFT57* | 6.88 | 2.17E-09 | 0.6695 | T | A | 0.0997 | A |
| 107,925,266 | rs9813446 | *IFT57* | 6.88 | 2.17E-09 | 0.6695 | A | G | 0.0998 | G |
| 107,925,686 | rs1289748 | *IFT57* | -6.86 | 2.45E-09 | -0.5890 | G | A | 0.8688 | G |
| 107,926,097 | rs1289747 | *IFT57* | -6.86 | 2.45E-09 | -0.5890 | A | G | 0.8689 | A |
| 107,926,126 | rs2701061 | *IFT57* | -6.86 | 2.45E-09 | -0.5890 | T | A | 0.8689 | T |
| 107,926,145 | rs1289746 | *IFT57* | -6.86 | 2.45E-09 | -0.5890 | T | A | 0.8689 | T |
| 107,926,258 | rs1289745 | *IFT57* | -6.86 | 2.45E-09 | -0.5890 | C | T | 0.8689 | C |
| 107,926,340 | rs1289744 | *IFT57* | -6.86 | 2.45E-09 | -0.5890 | C | T | 0.8689 | C |
| 107,926,518 | rs11924195 | *IFT57* | 6.88 | 2.17E-09 | 0.6695 | C | T | 0.0998 | T |
| 107,926,628 | rs11924228 | *IFT57* | 6.88 | 2.17E-09 | 0.6695 | C | T | 0.0998 | T |
| 107,926,759 | rs61705621 | *IFT57* | 6.88 | 2.17E-09 | 0.6695 | G | A | 0.0998 | A |
| 107,927,124 | rs57492931 | *IFT57* | 6.88 | 2.17E-09 | 0.6695 | T | A | 0.0998 | A |
| 107,927,244 | rs60037391 | *IFT57* | 6.88 | 2.17E-09 | 0.6695 | T | C | 0.0998 | C |
| 107,927,268 | rs57222320 | *IFT57* | 6.88 | 2.17E-09 | 0.6695 | T | C | 0.0998 | C |
| 107,927,420 | rs59142860 | *IFT57* | 6.88 | 2.17E-09 | 0.6695 | T | C | 0.0998 | C |
| 107,927,760 | rs1289743 | *IFT57* | -6.86 | 2.45E-09 | -0.5890 | C | G | 0.8688 | C |
| 107,928,040 | rs62267887 | *IFT57* | 6.88 | 2.17E-09 | 0.6695 | T | C | 0.0998 | C |
| 107,928,557 | rs35876429 | *IFT57* | -4.88 | 8.77E-05 | -0.2920 | C | T | 0.3522 | C |
| 107,929,140 | rs72933787 | *IFT57* | 6.88 | 2.17E-09 | 0.6695 | C | T | 0.0998 | T |
| 107,929,146 | rs72933788 | *IFT57* | 6.88 | 2.17E-09 | 0.6695 | G | T | 0.0998 | T |
| 107,929,228 | rs1289773 | *IFT57* | -6.86 | 2.49E-09 | -0.5883 | C | T | 0.8686 | C |
| 107,929,285 | rs112695180 | *IFT57* | 6.88 | 2.17E-09 | 0.6695 | G | T | 0.0998 | T |
| 107,929,699 | rs60731607 | *IFT57* | 6.88 | 2.17E-09 | 0.6695 | G | A | 0.0999 | A |
| 107,929,807 | rs1289772 | *IFT57* | -6.86 | 2.45E-09 | -0.5890 | C | A | 0.8688 | C |
| 107,929,821 | rs1289771 | *IFT57* | -6.86 | 2.45E-09 | -0.5890 | C | A | 0.8688 | C |
| 107,930,209 | rs11928402 | *IFT57* | 6.88 | 2.17E-09 | 0.6695 | C | T | 0.0999 | T |
| 107,930,210 | rs13094403 | *IFT57* | -4.88 | 8.77E-05 | -0.2920 | G | T | 0.3514 | G |
| 107,930,513 | rs1289769 | *IFT57* | -6.86 | 2.45E-09 | -0.5890 | C | T | 0.8687 | C |
| 107,930,578 | rs1289768 | *IFT57* | -6.86 | 2.45E-09 | -0.5890 | T | A | 0.8687 | T |
| 107,931,178 | rs62267890 | *IFT57* | 6.88 | 2.17E-09 | 0.6695 | C | T | 0.0999 | T |
| 107,931,574 | rs62267891 | *IFT57* | 6.88 | 2.17E-09 | 0.6695 | T | C | 0.0999 | C |
| 107,931,883 | rs72933794 | *IFT57* | 5.83 | 9.01E-07 | 0.6909 | T | C | 0.0691 | C |
| 107,931,977 | rs62267892 | *IFT57* | 6.88 | 2.17E-09 | 0.6695 | G | T | 0.0999 | T |
| 107,932,157 | rs75856459 | *IFT57* | 6.88 | 2.17E-09 | 0.6695 | A | G | 0.0999 | G |
| 107,932,180 | rs62267894 | *IFT57* | 6.88 | 2.17E-09 | 0.6695 | A | T | 0.0999 | T |
| 107,932,621 | rs62267895 | *IFT57* | 6.88 | 2.17E-09 | 0.6695 | G | A | 0.0999 | A |
| 107,933,433 | rs58424633 | *IFT57* | 6.88 | 2.17E-09 | 0.6695 | A | G | 0.0999 | G |
| 107,933,598 | rs56937459 | *IFT57* | 6.88 | 2.17E-09 | 0.6695 | A | T | 0.0999 | T |
| 107,933,793 | rs57362125 | *IFT57* | 6.88 | 2.17E-09 | 0.6695 | A | G | 0.0999 | G |
| 107,934,114 | rs62267897 | *IFT57* | 6.88 | 2.17E-09 | 0.6695 | A | T | 0.1000 | T |
| 107,934,302 | rs62267920 | *IFT57* | 6.88 | 2.17E-09 | 0.6695 | C | T | 0.1000 | T |
| 107,934,361 | rs62267921 | *IFT57* | 6.89 | 2.08E-09 | 0.6704 | G | A | 0.0999 | A |
| 107,934,412 | rs17829831 | *IFT57* | 6.89 | 2.08E-09 | 0.6704 | G | A | 0.0999 | A |
| 107,934,457 | rs62267922 | *IFT57* | 6.86 | 2.44E-09 | 0.6698 | A | G | 0.0996 | G |
| 107,934,886 | rs62267923 | *IFT57* | 6.88 | 2.17E-09 | 0.6695 | T | C | 0.1000 | C |
| 107,934,978 | rs72935713 | *IFT57* | 6.88 | 2.17E-09 | 0.6695 | T | C | 0.1000 | C |
| 107,935,543 | rs72935717 | *IFT57* | 6.88 | 2.17E-09 | 0.6695 | T | C | 0.0999 | C |
| 107,936,135 | rs1289765 | *IFT57* | -6.86 | 2.45E-09 | -0.5890 | T | C | 0.8686 | T |
| 107,936,641 | rs1289763 | *IFT57* | -6.86 | 2.45E-09 | -0.5890 | G | A | 0.8686 | G |
| 107,936,698 | rs11918055 | *IFT57* | 6.88 | 2.17E-09 | 0.6695 | T | C | 0.1000 | C |
| 107,937,165 | rs1289762 | *IFT57* | -6.86 | 2.45E-09 | -0.5890 | C | G | 0.8686 | C |
| 107,937,231 | rs1289761 | *IFT57* | -6.86 | 2.45E-09 | -0.5890 | T | C | 0.8686 | T |
| 107,937,360 | rs56273515 | *IFT57* | 6.88 | 2.17E-09 | 0.6695 | C | T | 0.1000 | T |
| 107,937,408 | rs1135897 | *IFT57* | 6.88 | 2.17E-09 | 0.6695 | C | T | 0.1000 | T |
| 107,937,571 | rs1289759 | *IFT57* | -6.86 | 2.45E-09 | -0.5890 | G | A | 0.8686 | G |
| 107,937,602 | rs1289758 | *IFT57* | -6.86 | 2.45E-09 | -0.5890 | C | T | 0.8686 | C |
| 107,937,942 | rs1289757 | *IFT57* | -6.86 | 2.45E-09 | -0.5890 | G | A | 0.8685 | G |
| 107,937,953 | rs1289756 | *IFT57* | -6.86 | 2.45E-09 | -0.5890 | G | C | 0.8685 | G |
| 107,938,060 | rs1289755 | *IFT57* | -6.86 | 2.46E-09 | -0.5890 | A | G | 0.8685 | A |
| 107,938,240 | rs1289754 | *IFT57* | -6.86 | 2.46E-09 | -0.5890 | G | C | 0.8685 | G |
| 107,938,872 | rs1289752 | *IFT57* | -6.86 | 2.46E-09 | -0.5890 | G | A | 0.8685 | G |
| 107,939,065 | rs1289751 | *IFT57* | -6.86 | 2.46E-09 | -0.5890 | A | T | 0.8685 | A |
| 107,939,225 | rs1034498 | *IFT57* | -6.86 | 2.46E-09 | -0.5890 | C | G | 0.8685 | C |
| 107,939,675 | rs1034497 | *IFT57* | 6.88 | 2.18E-09 | 0.6695 | T | C | 0.1001 | C |
| 107,939,784 | rs1034496 | *IFT57* | 6.88 | 2.18E-09 | 0.6695 | A | C | 0.1001 | C |
| 107,940,134 | rs57823884 | *IFT57* | 6.88 | 2.18E-09 | 0.6695 | A | G | 0.1001 | G |
| 107,940,458 | rs1289750 | *IFT57* | -6.86 | 2.46E-09 | -0.5890 | A | G | 0.8685 | A |
| 107,942,785 | rs62267926 | *IFT57* | 6.88 | 2.24E-09 | 0.6696 | G | A | 0.1001 | A |
| 107,946,831 | rs7627574 | *IFT57* | -4.69 | 1.97E-04 | -0.2896 | C | G | 0.3245 | C |
| 107,948,019 | rs1377276 | *IFT57* | 3.62 | 1.02E-02 | 0.2088 | C | G | 0.5585 | G |
| 107,950,892 | rs1289736 | *IFT57* | 3.60 | 1.12E-02 | 0.2068 | C | T | 0.5580 | T |
| 107,952,752 | rs1299075 | *IFT57* | 3.60 | 1.11E-02 | 0.2070 | A | C | 0.5586 | C |
| 107,955,515 | rs1580173 | *IFT57* | 3.89 | 4.25E-03 | 0.2242 | G | A | 0.5611 | A |
| 107,957,071 | rs13075733 | *IFT57* | 3.14 | 4.24E-02 | 0.1845 | G | T | 0.5852 | T |
| 107,957,436 | rs2966580 | *IFT57* | 3.73 | 7.24E-03 | 0.2249 | G | A | 0.6520 | A |
| 107,960,919 | rs16854357 | *IFT57* | 3.15 | 4.10E-02 | 0.1850 | G | A | 0.5853 | A |
| 107,970,160 | rs7636464 | *IFT57* | 3.19 | 3.75E-02 | 0.1878 | T | C | 0.5878 | C |
| 107,971,021 | rs7622810 | *IFT57* | 3.19 | 3.76E-02 | 0.1877 | T | C | 0.5878 | C |
| 107,976,756 | rs9831907 | *IFT57* | 3.18 | 3.80E-02 | 0.1875 | C | G | 0.5878 | G |
| 107,987,349 | rs75965832 | *IFT57* | 5.24 | 1.72E-05 | 0.6799 | C | T | 0.0593 | T |
| 107,989,949 | rs9876873 | *IFT57* | 3.22 | 3.44E-02 | 0.1889 | C | G | 0.5858 | G |
| 107,990,877 | rs13099083 | *IFT57* | 3.22 | 3.42E-02 | 0.1890 | A | T | 0.5858 | T |
| 107,996,184 | rs35360637 | *IFT57* | -4.03 | 2.61E-03 | -0.2577 | G | A | 0.3042 | G |
| 107,996,968 | rs13060852 | *IFT57* | -4.03 | 2.61E-03 | -0.2577 | A | G | 0.3043 | A |
| 107,997,514 | rs6437769 | *IFT57* | 3.22 | 3.42E-02 | 0.1890 | C | T | 0.5857 | T |
| 107,998,307 | rs2124735 | *IFT57* | 3.22 | 3.42E-02 | 0.1890 | C | T | 0.5857 | T |
| 108,000,268 | rs1975800 | *IFT57* | 3.22 | 3.42E-02 | 0.1890 | C | A | 0.5857 | A |
| 108,004,265 | rs7649599 | *IFT57* | 3.22 | 3.41E-02 | 0.1890 | G | A | 0.5856 | A |
| 108,011,037 | rs35746614 | *IFT57* | -3.98 | 3.06E-03 | -0.2540 | T | C | 0.3058 | T |
| 108,012,562 | rs2061246 | *IFT57* | 3.19 | 3.73E-02 | 0.1869 | C | T | 0.5842 | T |
| 108,013,801 | rs4855636 | *IFT57* | 3.19 | 3.73E-02 | 0.1869 | G | A | 0.5842 | A |
| 108,014,239 | rs113013592 | *IFT57* | -3.98 | 3.07E-03 | -0.2540 | A | C | 0.3058 | A |
| 108,015,247 | rs768342 | *IFT57* | 3.19 | 3.72E-02 | 0.1869 | G | T | 0.5842 | T |
| 108,015,911 | rs9829105 | *IFT57* | 3.19 | 3.71E-02 | 0.1870 | C | G | 0.5847 | G |
| 108,016,433 | rs9867495 | *IFT57* | 3.19 | 3.71E-02 | 0.1870 | A | T | 0.5847 | T |
| 108,019,057 | rs62266174 | *IFT57* | -3.99 | 2.98E-03 | -0.2548 | T | G | 0.3052 | T |
| 108,019,199 | rs17830546 | *IFT57* | -4.00 | 2.94E-03 | -0.2551 | A | C | 0.3054 | A |
| 108,022,663 | rs13099364 | *IFT57* | -4.03 | 2.57E-03 | -0.2576 | T | C | 0.3059 | T |
| 108,024,070 | rs752179 | *IFT57* | -4.00 | 2.91E-03 | -0.2558 | T | A | 0.3053 | T |
| 108,031,094 | rs1471093 | *IFT57* | 4.40 | 6.31E-04 | 0.2608 | G | A | 0.6151 | A |
| 108,036,819 | rs79269403 | *IFT57* | -3.59 | 1.15E-02 | -0.2537 | G | A | 0.2292 | G |
| 107,789,790 | rs56063635 | *MYH15* | -3.12 | 4.49E-02 | -0.2328 | T | A | 0.1919 | T |
| 107,823,224 | rs80003826 | *MYH15* | -3.92 | 3.80E-03 | -0.4498 | G | A | 0.0739 | G |
| 107,913,996 | rs79901199 | *MYH15* | -3.11 | 4.62E-02 | -0.3293 | A | G | 0.0896 | A |
| 108,103,427 | rs2290600 | *MYH15* | -3.24 | 3.24E-02 | -0.1904 | A | C | 0.4470 | A |
| 108,122,585 | rs1456721 | *MYH15* | -3.24 | 3.27E-02 | -0.1867 | C | T | 0.4972 | C |
| 108,122,595 | rs1456720 | *MYH15* | -3.24 | 3.25E-02 | -0.1868 | A | T | 0.4972 | A |
