# Supplementary material for: Admixture mapping reveals the association between Native American ancestry at 3q13.11 and reduced risk of Alzheimer’s disease in Caribbean Hispanics
Source: Alzheimers Res Ther. 2021 Jul 3;13:122. doi: 10.1186/s13195-021-00866-9 (PMC8254995; doi:10.1186/s13195-021-00866-9)
Supplement: Supplementary file 9 — Additional file 9: File format: Microsoft Word .docx file. Title: Evidence of differential gene expression between those with and without Alzheimer's disease for 3q13.11 candidate genes. Description: Data are stored in the Synapse repository, Synapse ID: syn11914606, https://www.synapse.org/#!Synapse:syn11914606, file meta.anlz.ad_cntrl.tsv, which included no values for CCDC54 or HHLA2. Legend: z.fixed = Z statistic for the fixed effects model, p.fixed = p-value for fixed effect model, z.random = Z statistic for the random effects model, p.random = p-value for the random effects model, fdr.fixed = false discovery rate from the fixed effects model, fdr.random = false discovery rate from random effects model. Results reaching the significance level of fdr.random < 0.05 are highlighted in bold. [file 13195_2021_866_MOESM9_ESM.docx]

**Additional File 9: Evidence of differential gene expression between those with and without Alzheimer's disease for 3q13.11 candidate genes.** Data are stored in the Synapse repository, Synapse ID: syn11914606, https://www.synapse.org/#!Synapse:syn11914606, file meta.anlz.ad_cntrl.tsv, which included no values for CCDC54 or HHLA2. Legend: z.fixed = Z statistic for the fixed effects model, p.fixed = p-value for fixed effect model, z.random = Z statistic for the random effects model, p.random = p-value for the random effects model, fdr.fixed = false discovery rate from the fixed effects model, fdr.random = false discovery rate from random effects model. Results reaching the significance level of fdr.random < 0.05 are highlighted in bold.

| **Gene** | **ensembl_gene_id** | **z.fixed** | **p.fixed** | **z.random** | **p.random** | **fdr.fixed** | **fdr.random** |
| --- | --- | --- | --- | --- | --- | --- | --- |
| ***ALCAM*** | ENSG00000170017 | 2.7537 | 5.89E-03 | **2.7537** | 5.89E-03 | 1.61E-02 | **2.76E-02** |
| ***BBX*** | ENSG00000114439 | 4.4301 | 9.42E-06 | **3.7336** | 1.89E-04 | 7.59E-05 | **1.84E-03** |
| *CBLB* | ENSG00000114423 | 1.6339 | 1.02E-01 | 0.9572 | 3.38E-01 | 1.71E-01 | 5.18E-01 |
| *CD47* | ENSG00000196776 | -3.2069 | 1.34E-03 | -2.2469 | 2.46E-02 | 4.68E-03 | 8.04E-02 |
| *IFT57* | ENSG00000114446 | 2.4699 | 1.35E-02 | 2.4699 | 1.35E-02 | 3.20E-02 | 5.11E-02 |
| *KIAA1524* | ENSG00000163507 | -3.8755 | 1.06E-04 | -1.9517 | 5.10E-02 | 5.68E-04 | 1.38E-01 |
| *MYH15* | ENSG00000144821 | -2.7070 | 6.79E-03 | -1.8357 | 6.64E-02 | 1.81E-02 | 1.67E-01 |
